# Sixteen New Prenylated Flavonoids from the Fruit of *Sinopodophyllum hexandrum*

**DOI:** 10.3390/molecules24173196

**Published:** 2019-09-03

**Authors:** Yanjun Sun, Haojie Chen, Junmin Wang, Meiling Gao, Chen Zhao, Ruijie Han, Hui Chen, Meng Li, Guimin Xue, Weisheng Feng

**Affiliations:** 1 Collaborative Innovation Center for Respiratory Disease Diagnosis and Treatment & Chinese Medicine, Development of Henan Province, Henan University of Chinese Medicine, Zhengzhou 450046, China; 2 School of Pharmacy, Henan University of Chinese Medicine, Zhengzhou 450046, China

**Keywords:** *Sinopodophyllum hexandrum*, prenylated flavonoid, cytotoxic activity

## Abstract

Sixteen new prenylated flavonoids, sinoflavonoids P–Z (**1**–**11**) and sinoflavonoids NA–NE (**12**–**16**), were isolated from the fruit of *Sinopodophyllum hexandrum*, along with eight known analogues (**17**–**24**). Their structures were elucidated on the basis of extensive spectroscopic data (HR-ESI-MS, ^1^H-NMR, ^13^C-NMR, HSQC, HMBC). The cytotoxic activities of compounds **1**–**18**, **20**, and **22** were evaluated by MTT assay. Compound **6** showed the most potent cytotoxicity in MCF-7, and HepG2 cell lines, with IC_50_ values of 6.25 and 3.83 μM, respectively.

## 1. Introduction

**Figure 1:**
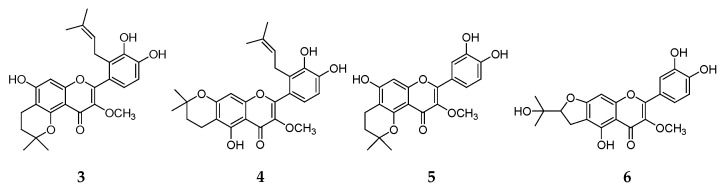

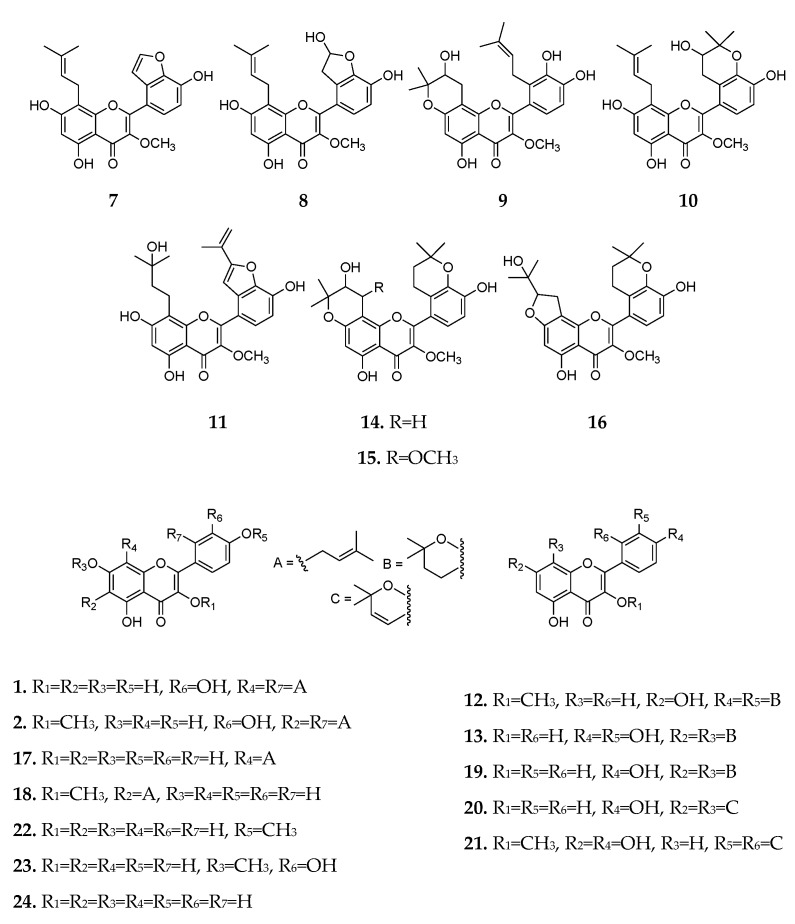
Structures of compounds **1**–**24.**

*Sinopodophyllum hexandrum*, belonging to the family of Berberidaceae, are widely distributed in the Southwest of China [1]. As an important medicinal plant, it was described in the Chinese Pharmacopoeia and in Tibetan medicine. The fruit of *S. hexandrum* is edible and popular with Tibetan people. It has been widely used in the treatment of amenorrhea, dead fetus, and placental retaining [2]. Previous phytochemical investigations on *S. hexandrum* allowed the isolation and identification of aryltetralin [1,3,4,5,6] and tetrahydrofuranoid lignans [7], flavonoids [2,8,9,10,11], labdane diterpenes [12], steroids [13], and phenolics [14]. As particularly rich in aryltetralin lactone lignans, the roots and rhizomes of the plant are mainly used for extracting podophyllotoxin, which is raw medicinal material for production of etoposide and teniposide [15]. However, its fruit is a rich source of prenylated flavonoids [2,10,11]. In our previous reports, 15 new prenylated flavonoids were found in the fruit of *S. hexandrum* [10,11]. As part of our continuous efforts toward discovering new cytotoxic natural products, 16 new prenylated flavonoids (**1**–**16**), together with eight known analogues (**17**–**24**), were isolated from the fruit of *S. hexandrum*. Details of the isolation, structure elucidation of all isolated compounds, as well as cytotoxicity of compounds **1**–**18**, **20**, and **22** against MCF-7 and HepG2 cell lines are described here (Figure 1).

## 2. Results and Discussion

The EtOH extract of the fruit of *S. hexandrum* was partitioned between petroleum ether (PE), CH_2_Cl_2_, EtOAc, *n*-BuOH and water, respectively. The EtOAc layer was fractionated and purified by repeated column chromatography, allowing the isolation of 24 flavonoids (**1**–**24**), including 16 new prenylated flavonoids, sinoflavonoids P–Z (**1**–**11**) and sinoflavonoids NA–NE (**12**–**16**), along with eight known analogues (**17**–**21**). By comparing their physical and spectroscopic data with literature values, the known metabolites were identified as 8-prenylkaempferol (**17**) [2], topazolin (**18**) [16], nor-β-anhydroicaritin (**19**) [17], citrusinol (**20**) [18], dysosmaflavone B (**21**) [19], 4′-methylkaempferol (**22**) [20], rhamnetin (**23**) [21], kaempferol (**24**) [8].

**Figure 2:**
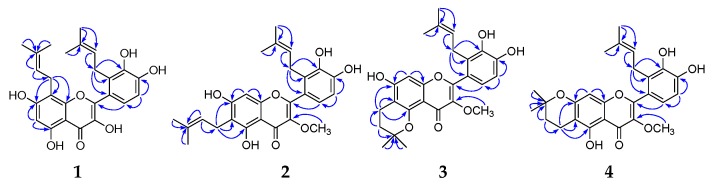

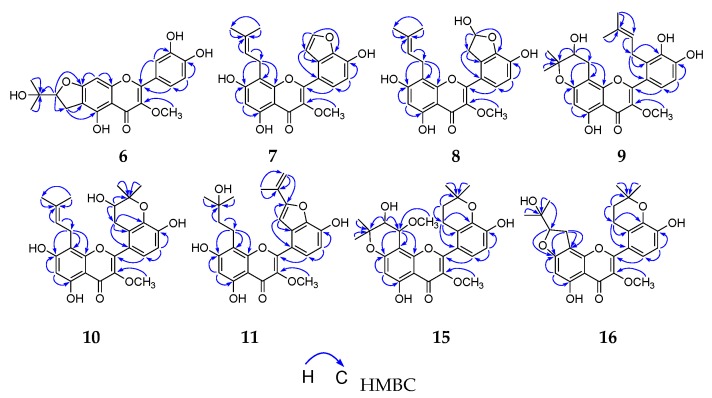
Key HMBC correlations of compounds **1**–**4**, **6**–**11**, **15**, **16.**

Compound **1** was obtained as a yellow, amorphous powder and possessed a molecular formula C_25_H_26_O_7_, as revealed from its HR-ESI-MS analysis (*m*/*z* 439.1760 [M + H]^+^, calcd 439.1757). The ^1^H-NMR spectrum (Table 1, see Figure S1) showed two aromatic systems including one 1,2,3,4-tetra-substituted benzene ring δ 6.74 (1H, d, *J* = 8.2 Hz) and 6.70 (1H, d, *J* = 8.2 Hz), one penta-substituted benzene ring δ 6.27 (1H, s), two 3-methyl-2-butenyls for two olefinic protons δ 4.94 (1H, t, *J* = 6.8 Hz) and 5.01 (1H, t, *J* = 6.8 Hz), four methyl groups δ 1.27 (3H, s), 1.39 (3H, s), 1.45 (3H, s) and 1.53 (3H, s), and two methylene groups δ 3.25 (2H, d, *J* = 6.8 Hz) and 3.21 (2H, d, *J* = 6.8Hz), and five phenolic hydroxyl groups δ 12.46 (1H, s), 10.64 (1H, s), 9.73 (1H, s), 8.77 (1H, s), and 8.42 (1H, s). The ^13^C-NMR spectrum (Table 2, see Figure S2) revealed a flavonol skeleton including one carbonyl group δ 176.5, two benzene rings, two oxygen-bearing olefinic carbons δ 150.7, 136.2, besides two 3-methyl-2-butenyls δ 20.9, 121.99, 130.6, 17.3, 25.4, 25.7, 122.04, 129.8, 17.2, 25.2. These spectroscopic data indicated that compound **1** was a prenylated flavonol derivative. The HMBC correlations (Figure 2) of methylene group δ 3.21 (2H, d, *J* = 6.8 Hz, H-1″) with C-7 (δ 160.8), C-8 (δ 105.4), and C-9 (δ 154.2), and δ 3.25 (2H, d, *J* = 6.8 Hz, H-1‴) with C-1′ (δ123.0), C-2′ (δ128.1), and C-3′ (δ143.0), indicated that two 3-methyl-2-butenyls were located at C-8 and C-2′, respectively. Thus, the structure of compound **1** was elucidated as 8,2′-di(3-methyl-2-butenyl)-5,7,3′,4′-tetrahydroxyflavone, and named sinoflavonoid P.

Compound **2** was obtained as a yellow, amorphous powder. The ^1^H-NMR (Table 1, see Figure S5) and ^13^C-NMR data (Table 2, see Figure S6) of compound **2** were closely correlated with those of **1**, but differed in the appearance of one methoxy group [δ 3.56 (3H, s), δ 60.3], and one olefinic carbon δ 93.3 at upper field instead of C-8 (δ 97.7) in **1**. The above data suggested that compound **2** was 6,2′-diprenyl-3-methoxyquercetin, which was also supported by HR-ESI-MS and the HMBC spectrum. The HR-ESI-MS gave an [M + H]^+^ ion peak at *m*/*z* 453.1910 (calcd 453.1913), being 14 mass units more than that of **1**. Two 3-methyl-2-butenyls were linked to C-6 and C-2′, respectively, due to the long range correlations of methylene group δ 3.21 (2H, d, *J* =7.1 Hz, H-1″) with C-5 (δ 158.5), C-6 (δ 111.1), and C-7 (δ 162.3), and of δ 3.24 (2H, d, *J* = 7.3 Hz, H-1‴) with C-1′ (δ 123.2), C-2′ (δ 128.2), and C-3′ (δ 143.7) in the HMBC spectrum (Figure 2). The methoxy group was located at C-3 by the HMBC correlation of the methoxy group δ 3.56 (3H, s) with C-3 (δ 139.0). Thus, the structure of compound **2** was elucidated as 6,2′-di(3-methyl-2-butenyl)-5,7,3′,4′-tetrahydroxy-3-methoxyflavone, and named sinoflavonoid Q.

Compounds **3** and **4** were obtained as yellow, amorphous powders. Their HR-ESI-MS showed the same molecular formula of C_26_H_28_O_7_, according to an [M + H]^+^ quasi-molecular ion peak (*m*/*z* 453.1910 (calcd 453.1913) in **3**; *m*/*z* 453.1912 (calcd 453.1913) in **4**). Their ^1^H (Table 1, see Figures S9 and S13) and ^13^C-NMR (Table 2, see Figures S10 and S14) spectra were similar to each other, and closely correlated with those of **2**, differing by the presence of one 2,2-dimethyldihydropyrano group instead of one 3-methyl-2-butenyl in **2**. One 2,2-dimethyldihydropyrano group was based on a series of signals consisting of two methylene groups (δ 2.54 (2H, t, *J* = 6.8 Hz), 1.74 (2H, t, *J* = 6.8 Hz), δ 16.7, 30.8 in **3**; δ 2.62 (2H, t, *J* = 6.7 Hz), 1.81 (2H, t, *J* = 6.7 Hz), δ 15.7, 30.9 in **4**), two tertiary-methyl groups (δ 1.30 (6H, s), δ 26.4 (×2) in **3**; δ 1.30 (6H, s), δ 26.3 (×2) in **4**), and one oxygen-bearing aliphatic quaternary carbon (δ 74.7 in **3**; δ76.2 in **4**). The methylene group (δ 2.54 (2H, t, *J* = 6.8 Hz, H-1″) in **3**; δ 2.62 (2H, t, *J* = 6.7 Hz, H-1″) in **4**) showed the long range correlations (Figure 2) with C-5 (δ 154.6 in **3**; δ 158.2 in **4**), C-6 (δ 105.0 in **3**; δ 104.4 in **4**), and C-7 (δ 159.8 in **3**; δ 159.8 in **4**), indicating that the 2,2-dimethyldihydropyrano group was attached to C-6 and C-7, or C-5 and C-6. The presence of the phenolic hydroxyl group (δ 10.60 (1H, s, 7-OH) in **3**; δ 13.00 (1H, s, 5-OH) in **4**) supported the linking position of the 2,2-dimethyldihydropyrano group (C-5 and C-6 in **3**; C-6 and C-7 in **4**). Thus, the structures of compounds **3** and **4** were deduced respectively as 5,6-(2,2-dimethyldihydropyrano)-2′-(3-methyl-2-butenyl)-7,3′,4′-trihydroxy-3-methoxyflavone (**3**) and 6,7-(2,2-dimethyldihydropyrano)-2′-(3-methyl-2-butenyl)-5,3′,4′-trihydroxy-3-methoxyflavone (**4**), and named sinoflavonoids R and S.

Compound **5** was obtained as a yellow, amorphous powder. Its ^1^H (Table 1, see Figure S17) and ^13^C-NMR spectra (Table 2, see Figure S18) was quite similar to those of **3**, except for the appearance of another aromatic proton δ 7.46 (1H, d, *J* = 2.2 Hz) instead of the 3-methyl-2-butenyl in **3**, suggesting the absence of 3-methyl-2-butenyl at C-2′ in **5**. This was further supported by the HR-ESI-MS of **5** which gave an [M + H]^+^ ion peak at *m*/*z* 385.1262 (calcd 385.1287), being 68 mass units less than that of **3**. Thus, compound **5** was deduced as 5,6-(2,2-dimethyldihydropyrano)-7,3′,4′-trihydroxy-3-methoxyflavone, and named sinoflavonoid T.

Compound **6** was obtained as a yellow, amorphous powder. The molecular formula was found to be C_21_H_20_O_8_, as deduced by analysis of the [M + Na]^+^ molecular ion peak at *m*/*z* 423.1056 (calcd 423.1056) in the HR-ESI-MS. Its ^1^H (Table 1, see Figure S21) and ^13^C-NMR spectra (Table 2, see Figure S22) were similar to those of **5**, except that one 2-(1-hydroxy-1-methylethyl)dihydrofurano group was observed instead of one 2,2-dimethyldihydropyrano group in **5**. 2-(1-Hydroxy-1-methylethyl)dihydrofurano group was deduced by the HMBC correlations (Figure 2) from two tertiary-methyls δ1.13 (3H, s, H-4″), 1.14 (3H, s, H-5″) to C-3″ (δ 70.0), C-2″ (δ 91.5), from oxymethine group δ 4.75 (1H, t, *J* = 8.4 Hz, H-2″) to C-1″ (δ 25.7), C-3″ (δ 70.0), C-4″ (δ 24.8), and C-5″ (δ 25.9), from methylene group δ 3.06 (2H, d, *J* = 8.4 Hz, H-1″) to C-2″ (δ 91.5). 2-(1-Hydroxy-1-methylethyl)dihydrofurano group was located at C-6 and C-7 from the HMBC correlations of oxymethine group δ 4.75 (1H, t, *J* = 8.4 Hz, H-2″) with C-7 (δ 166.2), of methylene group δ 3.06 (2H, d, *J* = 8.4 Hz, H-1″) with C-5 (δ 155.3), C-6 (δ 108.8), C-7 (δ 166.2), and in combination with the chelated phenolic hydroxyl group δ 12.92 (1H, s, 5-OH). Thus, compound **6** was deduced as 6,7-[2-(1-hydroxy-1-methylethyl)dihydrofurano]-5,3′,4′-trihydroxy-3-methoxyflavone, and named sinoflavonoid U.

Compound **7** was obtained as a yellow, amorphous powder and possessed a molecular forma of C_23_H_20_O_7_, derived from its HR-ESI-MS analysis (*m*/*z* 407.1111 [M−H]^−^, calcd C_23_H_19_O_7_, 407.1131). The ^1^H (Table 1, see Figure S25) and ^13^C-NMR spectra (Table 2, see Figure S26) of compound **7** was quite similar to those of **1**, except for the appearance of one methoxy group and one disubstituted furan ring for two olefinic protons δ 7.05 (1H, d, *J* = 2.1 Hz, H-3‴) and 8.09 (1H, d, *J* = 2.1 Hz, H-2‴) instead of one 3-methyl-2-butenyl in **1**. This was also further supported by HMBC spectrum (Figure 2). The olefinic protons δ 7.05 (1H, d, *J* = 2.1 Hz, H-1‴) and 8.09 (1H, d, *J* = 2.1 Hz, H-2‴) showed the HMBC correlations with C-1′ (δ 113.4), C-2′ (δ 128.1), C-3′ (δ 143.1), and C-2′ (δ 128.1), C-3′ (δ 143.1), respectively, indicating the disubstituted furan ring was attached to C-2′ and C-3′. The methoxy group was located at C-3 by the HMBC correlation of the methoxy group δ 3.65 (3H, s) with C-3 (δ 137.7). Thus, compound **7** was deduced as 2′,3′-furano-5,3′,4′-trihydroxy-3-methoxyflavone, and named as sinoflavonoid V.

Compound **8** was obtained as a yellow, amorphous powder. Its ^1^H (Table 1, see Figure S29) and ^13^C-NMR spectra (Table 2, see Figure S30) were quite similar to those of **7**, except that one 2-hydroxydihydrofuran ring was observed instead of one furan ring in **7**. The 2-hydroxydihydrofuran ring was determined by one methylene group δ 3.44 (1H, dd, *J* = 17.1, 6.8 Hz), 2.95 (1H, dd, *J* = 17.1, 2.4 Hz), δ 38.2, one dioxymethine group δ 6.03 (1H, br.s), δ 101.0. Those were further supported by its HR-ESI-MS, which gave an [M + H]^+^ quasi-molecular ion peak *m*/*z* 427.1390 (calcd 427.1393), being 18 mass units more than that of **7**. Thus, compound **8** was identified as 8-(3-methyl-2-butenyl)-2′,3′-(2-hydroxydihydrofurano)-5,7,4′-trihydroxy-3-methoxyflavone, and named as sinoflavonoid W.

Compounds **9** and **10** were obtained as yellow, amorphous powders. Their molecular formulae were assigned as C_26_H_28_O_8_ by HR-ESI-MS (*m*/*z* 491.1683 [M + Na]^+^ (calcd 491.1682) in **9**; *m*/*z* 491.1669 [M + Na]^+^ (calcd 491.1682) in **10**). Their ^1^H (Table 1, see Figures S33 and S37) and ^13^C-NMR spectra (Table 2, see Figures S34 and S38) were similar to those of **8**. Two tertiary-methyl signals (δ 1.27 (3H, s), 1.20 (3H, s), δ 25.1, 20.8 in **9**; δ 1.32 (3H, s), 1.17 (3H, s), δ 25.3, 19.8 in **10**), one methylene group (δ 2.79 (1H, dd, *J* = 16.5, 5.2 Hz), 2.47 (1H, dd, *J* = 16.5, 7.1 Hz), δ 24.9 in **9**; δ 2.75 (1H, dd, *J* = 17.0, 5.4 Hz), 2.55 (1H, dd, *J* = 17.0, 8.2 Hz), δ 29.5 in **10**), and one oxymethine group (δ 3.67 (1H, dd, *J* = 7.1, 5.2 Hz), δ 66.8 in **9**; δ 3.60 (1H, dd, *J* = 8.2, 5.4 Hz), δ 67.8 in **10**) were observed, implying the presence of one 2,2-dimethyl-3-hydroxydihydropyrano group in **9** and **10** instead of 2-hydroxydihydrofurano ring in **8**, respectively. The 2,2-dimethyl-3-hydroxydihydropyrano group and 3-methyl-2-butenyl in **9** were attached to C-7 and C-8, and C-2′ and C-3′, respectively, by the HMBC correlations (Figure 2) from H-1″ (δ 2.79, 2.47) to C-7 (δ 158.7), C-8 (δ 99.0), and C-9 (δ 154.1), and from H-1‴ (δ 3.23) to C-1′ (δ 121.2), C-2′ (δ 127.7), and C-3′ (δ 143.3). In contrast, the 3-methyl-2-butenyl and 2,2-dimethyldihydropyrano group in **10** was locatedat C-8, and C-2′, C-3′, respectively, by the HMBC correlations of H-1″ (δ 3.23) with C-7 (δ 161.5), C-8 (δ 105.9) and C-9 (δ 154.1), and of H-1‴ (δ 2.75, 2.55) with C-1′ (δ 120.5), C-2′ (δ 120.6) and C-3′ (δ 141.0). Thus, compounds **9** and **10** were elucidated respectively as 7,8-(2,2-dimethyldihydropyrano)-2′-(3-methyl-2-butenyl)-5,3′,4′-trihydroxy-3-methoxyflavone (**9**), 8-(3-methyl-2-butenyl)-2′,3′-(2,2-dimethyldihydropyrano)-5,7,4′-trihydroxy-3-methoxyflavone (**10**), and named sinoflavonoids X and Y.

Compound **11** was obtained as a yellow, amorphous power and possessed a molecular forma of C_26_H_26_O_8_, derived from its HR-ESI-MS analysis (*m*/*z* 465.1544 [M − H]^−^, calcd C_26_H_25_O_8_, 465.1549). Its ^1^H-NMR (Table 1, see Figure S41) and ^13^C NMR data (Table 2, see Figure S42) were closely correlated with those of **7**, but differed in the appearance of an isopropenyl and 3-hydroxy-3-methylbutyl in **11** instead of an olefinic proton δ 8.09 (1H, d, *J* = 2.1 Hz) and 3-methyl-2-butenyl in **7**, respectively. The isopropenyl was deduced by two olefinic protons δ 5.77 (1H, s), 5.28 (1H, s), one tertiary-methyl group δ 2.10 (3H, s), δ 18.9, two olefinic carbons δ 114.1, 132.5. The furan ring was inferred from one olefinic proton δ 7.01 (1H, s), and two characteristic olefinic carbons δ 104.2, 156.5. In the HMBC spectrum (Figure 2), the long range correlations of the olefinic protons δ 5.77 (1H, s), 5.28 (1H, s), the methyl group 2.10 (3H, s) with the olefinic carbon δ 156.5 (C-2‴) indicated the isopropenyl was linked to C-2‴. One 3-hydroxy-3-methylbutyl was based on a series of signals consisting of two methylene groups δ 2.67 (2H, m), 1.47 (2H, m), δ 17.5, 47.8, two tertiary-methyl groups δ 0.98 (6H, s), δ 28.8 (×2), and one oxygen-bearing aliphatic quaternary carbon δ 68.7. The HMBC spectrum also showed the long range correlations of the methylene protons δ 2.67 (2H, m, H-1″) with C-7 (δ 161.7), C-8 (δ 107.2), and C-9 (δ 154.1), indicating that 3-hydroxy-3-methylbutyl was attached to C-8. Thus, compound **11** was elucidated as 8-(3-hydroxy-3-methylbutyl)-2′,3′-(2-isopropenylfurano)-5,7,4′-trihydroxy-3-methoxyflavone, and named sinoflavonoid Z.

Compound **12** was obtained as a yellow, amorphous powder and possessed a molecular forma of C_21_H_20_O_6_, derived from its HR-ESI-MS analysis (*m*/*z* 369.1336 [M + H]^+^, calcd C_21_H_21_O_6_, 369.1338). Its ^1^H and ^13^C-NMR spectra were similar to kaempferol [8], except for the appearance of 2,2-dimethyldihydropyrano group and one methoxy group in **12**. 2,2-Dimethyldihydropyrano group was proved by two tertiary-methyl signals δ1.32 (6H, s), δ 27.0 (×2), two methylene groups δ 1.82 (2H, t, *J* = 6.5 Hz), 2.83 (2H, t, *J* = 6.5 Hz), δ 32.5, 22.2, and one oxygen-bearing aliphatic quaternary carbon δ 75.8. By the HMBC correlations (Figure 2) of δ 2.83 (2H, t, *J* = 6.5Hz, H-1″) with C-2′ (δ 130.2), C-3′ (δ 121.5) and C-4′ (δ 156.8), 2,2-dimethyldihydropyrano group was linked to C-3′ and C-4′. The methoxy group was located at C-3, based on the HMBC correlation between the methoxy group δ 3.78 (3H, s) and C-3 (δ 138.2). Thus, compound **12** was elucidated as 3′,4′-(2,2-dimethyldihydropyrano)-5,7-dihydroxy-3-methoxyflavone, and named sinoflavonoid NA.

Compound **13** was obtained as a yellow, amorphous powder and possessed the molecular formula C_20_H_18_O_7_, as revealed from its HR-ESI-MS analysis (*m*/*z* 371.0989 [M + H]^+^, calcd C_20_H_19_O_7_, 371.1131). Its ^1^H (Table 1, see Figure S49) and ^13^C-NMR spectra (Table 2, see Figure S50) were similar to quercetin [8], except for the appearance of 2,2-dimethyldihydropyrano group in **13**. 2,2-Dimethyldihydropyrano group was proved by two tertiary-methyl signals δ 1.32 (6H, s), δ 26.3 (×2), two methylene groups δ 1.87 (2H, t, *J* = 6.6 Hz), 2.84 (2H, t, *J* = 6.6Hz), δ 15.7, 30.9, and one oxygen-bearing aliphatic quaternary carbon δ 76.2. By the HMBC correlations (Figure 2) of δ 2.84 (2H, t, *J* = 6.6Hz, H-1″) with C-7 (δ 159.1), C-8 (δ 99.7) and C-9 (δ 153.0), 2,2-dimethyldihydropyrano group was linked to C-7 and C-8. Thus, compound **13** was deduced as 7,8-(2,2-dimethyldihydropyrano)-5,3,3′,4′-tetrahydroxyflavone, named sinoflavonoid NB.

Compound **14** was obtained as a yellow, amorphous powder. Its ^1^H (Table 1, see Figure S53) and ^13^C-NMR spectra (Table 2, see Figure S54) were quite similar to those of **9**, respectively, except for the observation of 2,2-dimethyldihydropyrano group in **14** instead of 3-methyl-2-butenyl in **9**. This was further confirmed by their HR-ESI-MS, which gave the same molecular formula C_26_H_28_O_8_ by the quasi-molecular ion peak (*m*/*z* 469.1853 [M + H]^+^ (calcd 469.1862) in **14**, *m*/*z* 491.1682 [M + Na]^+^ in **9**). The 2,2-dimethyldihydropyrano group was attached to C-2′ and C-3′, by the HMBC correlations (Figure 2) of the methylene protons δ 2.68 (2H, t, *J* = 6.6 Hz, H-1‴) with C-1′ (δ 120.3), C-2′ (δ 121.3), and C-3′ (δ 142.0). Thus, compound **14** was elucidated as 7,8-bis-2′,3′-(2,2-dimethyldihydropyrano)-5,4′,2″-trihydroxy-3-methoxyflavone, and named sinoflavonoid NC.

Compound **15** was obtained as a yellow, amorphous powder. Its ^1^H (Table 1, see Figure S57) and ^13^C-NMR spectra (Table 2, see Figure S58) were quite similar to those of **14**, respectively, except for the observation of one methoxy group δ 3.31 (3H, s), δ 56.9 in **15**, suggesting **15** to be a further methyl ether derivative of **14**. This was further confirmed by the HR-ESI-MS, which gave the molecular formula C_27_H_30_O_9_ by the quasi-molecularion peak *m*/*z* 521.1792 [M + Na]^+^ (calcd 521.1788), being 14 mass units more than that of **14**. In the HMBC spectrum (Figure 2), the additional methoxy group was located at C-1″, based on the long range correlation between the methoxy group δ 3.31 (3H, s) and C-1″ (δ 73.9). Thus, compound **15** was elucidated as 7,8-bis-2′,3′-(2,2-dimethyldihydropyrano)-5,4′,2″-trihydroxy-3,1″-dimethoxyflavone, and named sinoflavonoid ND.

Compound **16** was obtained as a yellow, amorphous powder. Its ^1^H (Table 1, see Figure S61) and ^13^C-NMR spectra (Table 2, see Figure S62) were quite similar to those of **14**, except for the appearance of one 2-(1-hydroxy-1-methylethyl)dihydrofurano group in **16** instead of 3-hydroxy-2,2-dimethyldihydropyrano group in **14**. 2-(1-Hydroxy-1-methylethyl)dihydrofurano group was deduced by the HMBC correlations (Figure 2) from two tertiary-methyls δ1.13 (3H, s, H-4″), 1.14 (3H, s, H-5″) to C-3″ (δ 69.9), C-2″ (δ 91.5), from oxymethine group δ 4.74 (1H, t, *J* = 8.5 Hz, H-2″) to C-1″ (δ 25.2), C-3″ (δ 69.9), C-4″ (δ 24.8), and C-5″ (δ 25.7), from methylene group δ 3.13 (2H, d, *J* = 8.5 Hz, H-1″) to C-2″ (δ 91.5). 2-(1-Hydroxy-1-methylethyl)dihydrofurano group was located at C-7 and C-8, based on the HMBC correlation of methylene group δ 3.13 (2H, d, *J* = 8.5 Hz) with C-7 (δ 166.2), C-8 (δ 104.4), C-9 (δ 151.3). Thus, compound **16** was deduced as 7,8-[2-(1-hydroxy-1-methylethyl)dihydrofurano]-2′,3′-(2,2-dimethyldihydropyrano)-5,4′-dihydroxy-3-methoxyflavone, and named sinoflavonoid NE.

Compounds **1**–**18**, **20**, **22** were tested for their in vitro cytotoxic activities against MCF-7 and HepG2 cell lines using the MTT assay [11], with 5-fluorouracil as a positive control, and IC_50_ values were summarized in Table 3. Among the tested compounds, only compound **6** exhibited the most potent cytotoxic activities against MCF-7 and HepG2 cell lines, with an IC_50_ value of 6.25 and 3.83*μ*M, respectively. Compound **6** was more cytotoxic than 5-fluorouracil, whereas compounds **5** displayed no cytotoxicity against MCF-7 and HepG2 cell lines. Compound **5** has the same B and C rings from flavone skeleton as **6**, so the variation in cytotoxicity between them indicates 2-(1-hydroxy-1-methylethyl)dihydrofurano group on ring A is structurally required for the cytotoxity against the MCF-7 and HepG2 cells lines. Furthermore, the cytotoxic activity may be affected by the position of furano or dihydrofurano group on the ring A, which needs to be verified with more similar derivatives. With the promising cytotoxicities against two cell lines, compound **6** may be the optimal lead compound for structure optimization studies.

## 3. Materials and Methods

### 3.1. General Experimental Procedures

The UV spectra were measured on a Shimadzu UV-1700 spectrometer (Shimadzu Corporation, Kyoto, Japan). The IR spectra were measured on a Nicolet 10 Microscope Spectrometer (Thermo Scientific, San Jose, CA, USA). The 1D and 2D-NMR spectra were recorded on Bruker-AC (E)-500 spectrometer (Bruker AM 500, Fällanden, Switzerland) using tetramethylsilane (TMS) as an internal standard. The HR-ESI-MS was determined on a Bruker microTOF-Q instrument (Bruker BioSpin, Rheinstetten, Germany). Column chromatography was performed with silica gel (200–300 mesh; Qingdao Marine Chemical Inc., Qingdao, China), sephadex LH-20 (GE Healthcare), and ODS (50 µm; YMC Co. LTD., Kyoto, Japan). Preparative high performance liquid chromatography (HPLC) separations were performed on a SEP system (Beijing Sepuruisi scientific Co., Ltd., China) equipped with a variable-wavelength UV detector, using a YMC-Pack ODS-A column (250 × 20 mm, 5 μm). Chemical reagents for isolation were of analytical grade and purchased from Tianjin Siyou Co., Ltd., China. Biological reagents were from Sigma Company. Human heptocellular (HepG2), and breast (MCF-7) cancer cell lines were from Institute of Materia Medica, Chinese Academy of Medical Sciences and Peking Union Medical College, China.

### 3.2. Plant Material

The plant material was collected from Deqin, Yunnan Province, China, in September 2013, and identified by Prof. Chengming Dong as the fruit of *S. hexandrum*. A voucher specimen (SE 20130929) was deposited at the School of Pharmacy, Henan University of Traditional Chinese Medicine.

### 3.3. Extraction and Isolation

The powered fruit of *S. hexandrum* (9.1 kg) were refluxed with 95% EtOH three times (each, 2h, 20L). The filtrate was concentrated under reduced pressure to yield a dark brown residue (1.6 kg). The residue was suspended in water and partitioned with petroleum ether (PE), CH_2_Cl_2_, EtOAc, and *n*-BuOH, successively.

The EtOAc layer (142.71 g) was fractionated by silica gel column chromatography (CC, 100 × 10 cm) with a gradient of PE (60–90 ℃)–acetone. Sixteen fractions E1–E16 were obtained on the basis of TLC monitoring. Fraction E7 (4.79 g) was chromatographed over open ODS (50 × 2 cm) eluted with a gradient of methanol–H_2_O (*v*/*v* 60:40, 70:30, 80:20, 90:10) to obtain sub-fractions E7-1~E7-6. Sub-fraction E7-4 (1.62 g) was further purified by silica gel CC (20 × 1 cm) eluted with PE-acetone (100:10, 100:30, 100:50) to give **12** (4.8 mg), **17** (53.8 mg), **18** (6.4 mg), **19** (2.6 mg), **20** (1.9 mg). Fraction E8 (5.02 g) was subjected to sephadex LH-20 CC (90 × 2.5 cm) eluted by methanol to yield sub-fractions E8-1~E8-5. Sub-fraction E8-4 (1.03 g) was further submitted to preparative HPLC eluted with methanol-H_2_O (72: 28) at a flow rate of 7 mL/min to obtain **10** (1.8 mg, t_R_ 25 min), **2** (5.9 mg, t_R_ 36 min). Sub-fraction E8-5 (1.75 g) was further applied to preparative HPLC eluted with methanol-H_2_O (75:25) at a flow rate of 7 mL/min to give **24** (30.8 mg, t_R_ 16 min), **23** (1.8 mg, t_R_ 19 min), **22** (2.6 mg, t_R_ 27 min), **21** (2.2 mg, t_R_ 32 min). Fraction E9 (2.45 g) was separated by silica gel CC eluted by PE-acetone (100:30) to give **5** (6.2 mg) and **11** (4.9 mg). Fraction E10 (2.05 g) was submitted to sephadex LH-20 CC (1.5 × 50 cm) eluted by methanol to yield sub-fractions E10-1~E10-3. Sub-fraction E10-2 was purified by preparative HPLC eluted with MeOH-H_2_O (69:21) at 7 mL/min to yield **6** (8.5 mg, t_R_ 42 min), **16** (7.6 mg, t_R_ 62 min), **14** (5.3 mg, t_R_ 66 min). Fraction E11 (1.95 g) was applied to silica gel CC (45 × 2 cm) eluted by PE-acetone (100:7, 100:10, 100:15, 100: 20) to yield sub-fractions E11-1~E11-4. Sub-fraction E11-4 was further purified by preparative HPLC eluted with methanol-H_2_O (80:20) at a flow rate of 7 mL/min to give **15** (4.5 mg, t_R_ 21 min), **4** (3.7 mg, t_R_ 69 min). Fraction E12 (1.70 g) was chromatographed over open ODS (25 × 2 cm) eluted with a gradient of methanol–H_2_O (50:50, 70:30, 80:20) to yield sub-fractions E12-1~E12-3. Sub-Fraction E12-3 (0.5 g) was further purified by preparative HPLC eluted with MeOH–H_2_O (60:40) at 7 mL/min to yield **1** (13.5 mg, t_R_ 26 min), **7** (8.5 mg, t_R_ 40 min). Fraction E13 (2.58 g) was subjected to silica gel CC (35 × 2 cm) eluted by PE-acetone (100:40) to give **3** (4.8 mg) and **13** (5.2 mg). Fraction E14 (1.73 g) was submitted to sephadex LH-20 CC (60 × 2.5 cm) eluted by methanol to yield sub-fractions E14-1~E14-3. Sub-fraction E14-3 was subjected to preparative HPLC eluted with methanol–H_2_O (75:25) at a flow rate of 7 mL/min to give **9** (2.7 mg, t_R_ 16 min), **8** (3.2 mg, t_R_ 25 min).

### 3.4. Spectroscopic and Physical Data

Sinoflavonoid P (**1**): yellow, amorphous powder; UV (MeOH) λ_max_ (log ε) 260 (0.40), 364 (0.22) nm; IR*ν*_max_ 3356, 2924, 2854, 1651, 1603, 1560, 1511, 1423, 1364, 1316, 1260, 1211, 1188, 1149, 1112 cm^−1^; HR-ESI-MS (positive): *m*/*z* 439.1760 [M + H]^+^ (calcd. for C_25_H_27_O_7_, 439.1757), *m*/*z* 461.1576 [M + Na]^+^ (calcd. for C_25_H_26_O_7_Na, 461.1576); NMR data (DMSO-*d*_6_), see Table 1; Table 2.

Sinoflavonoid Q (**2**): yellow, amorphous powder; UV (MeOH) λmax (log ε) 262 (1.24), 321 (0.73) nm; IR*ν*_max_ 3354, 2961, 2926, 2855, 1659, 1611, 1573, 1468, 1358, 1293, 1234, 1155, 1092 cm^−1^; HR-ESI-MS (positive): *m*/*z* 453.1910 [M + H]^+^ (calcd. for C_26_H_29_O_7_, 453.1913); NMR data (DMSO-*d*_6_), see Table 1; Table 2.

Sinoflavonoid R (**3**): yellow, amorphous powder; UV (MeOH) λmax (log ε) 262 (1.24), 321 (0.73) nm; IR*ν*_max_ 3374, 2959, 2926, 2854, 1650, 1597, 1451, 1359, 1293, 1228, 1162, 1092 cm^−1^; HR-ESI-MS (positive): *m*/*z* 453.1910 [M + H]^+^ (calcd. for C_26_H_29_O_7_, 453.1913); NMR data (DMSO-*d*_6_), see Table 1; Table 2.

Sinoflavonoid S (**4**): yellow, amorphous powder; UV (MeOH) λmax (log ε) 261 (1.24), 325 (0.60) nm; IR*ν*_max_ 3524, 2975, 2930, 2856, 1651, 1604, 1462, 1373, 1301, 1258, 1161, 1091 cm^−1^; HR-ESI-MS (positive): *m*/*z* 453.1912 [M + H]^+^ (calcd. for C_26_H_29_O_7_, 453.1913); NMR data (DMSO-*d*_6_), see Table 1;Table 2.

Sinoflavonoid T (**5**): yellow, amorphous powder; UV (MeOH) λmax (log ε) 256 (0.66), 345 (0.75) nm; IR*ν*_max_ 3421, 2974, 2941, 1655, 1617, 1571, 1454, 1287, 1241, 1159, 1088 cm^−1^; HR-ESI-MS (positive): *m*/*z* 385.1262 [M + H]^+^ (calcd. for C_21_H_21_O_7_, 385.1287), (negative): *m*/*z* 383.1116 [M − H]^−^ (calcd. for C_21_H_19_O_7_, 383.1131); NMR data (DMSO-*d*_6_), see Table 1; Table 2.

Sinoflavonoid U (**6**): yellow, amorphous powder; [α${]}_{D}^{25}$ − 13.7 (c 0.13, MeOH); UV (MeOH) λmax (log ε) 258 (0.48), 354 (0.56) nm; IR*ν*_max_ 3393, 2925, 1656, 1588, 1455, 1341, 1239, 1159, 1088 cm^−1^; HR-ESI-MS (positive): *m*/*z* 423.1056 [M + Na]^+^ (calcd. for C_21_H_20_O_8_Na, 423.1056), (negative): *m*/*z* 399.1084 [M − H]^−^ (calcd. for C_21_H_19_O_8_, 399.1080); NMR data (DMSO-*d*_6_), see Table 1; Table 2.

Sinoflavonoid V (**7**): yellow, amorphous powder; UV (MeOH) λmax (log ε) 269 (0.11), 359 (0.07) nm; IR*ν*_max_ 3330, 2957, 2925, 2870, 2855, 1650, 1610, 1562, 1511, 1496, 1453, 1424, 1364, 1334, 1307, 1230, 1154, 1098 cm^−1^; HR-ESI-MS (positive): *m*/*z* 409.1282 [M + H]^+^ (calcd. for C_23_H_21_O_7_, 409.1287), (negative): *m*/*z* 407.1111 [M − H]^−^ (calcd. for C_23_H_19_O_7_, 407.1131); NMR data (DMSO-*d*_6_), see Table 1; Table 2.

Sinoflavonoid W (**8**): yellow, amorphous powder; [α${]}_{D}^{25}$–11.5 (c 0.09, MeOH); UV (MeOH) λmax (log ε) 264 (0.39), 350 (0.15) nm; IR*ν*_max_ 3350, 2966, 2926, 2855, 1651, 1610, 1506, 1448, 1362, 1306, 1228, 1069 cm^−1^; HR-ESI-MS (positive): *m*/*z* 427.1390 [M + H]^+^ (calcd. for C_23_H_23_O_8_, 427.1393), *m*/*z* 449.1210 [M + Na]^+^ (calcd. for C_23_H_22_O_8_Na, 449.1212); NMR data (DMSO-*d*_6_), see Table 1; Table 2.

Sinoflavonoid X (**9**): yellow, amorphous powder; [α${]}_{D}^{25}$–8.9 (c 0.04, MeOH); UV (MeOH) λmax (log ε) 264 (0.41), 350 (0.18) nm; IR*ν*_max_ 3368, 2982, 2926, 2854, 1655, 1597, 1506, 1486, 1446, 1355, 1297, 1232, 1172,1149, 1033 cm^−1^; HR-ESI-MS (positive): *m*/*z* 491.1683 [M + Na]^+^ (calcd. for C_26_H_28_O_8_Na, 491.1682); NMR data (DMSO-*d*_6_), see Table 1; Table 2.

Sinoflavonoid Y (**10**): yellow, amorphous powder; [α${]}_{D}^{25}$–7.4 (c 0.03, MeOH); UV (MeOH) λmax (log ε) 264 (0.38), 350 (0.13) nm; IR*ν*_max_ 3340, 2982, 2930, 2854, 1651, 1584, 1490, 1449, 1359, 1302, 1227, 1171, 1145, 1067 cm^−1^; HR-ESI-MS (positive): *m*/*z* 491.1669 [M + Na]^+^ (calcd. for C_26_H_28_O_8_Na, 491.1682); NMR data (DMSO-*d*_6_), see Table 1; Table 2.

Sinoflavonoid Z (**11**): yellow, amorphous powder; UV (MeOH) λmax (log ε) 263 (0.14), 332 (0.09) nm; IR*ν*_max_ 3403, 2957, 2922, 2851, 1654, 1578, 1488, 1457, 1375, 1361, 1302, 1229, 1161, 1076 cm^−1^; HR-ESI-MS (positive): *m*/*z* 465.1544 [M − H]^−^ (calcd. for C_26_H_25_O_8_, 465.1549); NMR data (DMSO-*d*_6_), see Table 1; Table 2.

Sinoflavonoid NA (**12**): yellow, amorphous powder; UV (MeOH) λmax (log ε) 268 (0.53), 355 (0.60) nm; IR*ν*_max_ 3421, 2974, 2929, 2853, 1647, 1603, 1507, 1489, 1448, 1358, 1306, 1160, 1089 cm^−1^; HR-ESI-MS (positive): *m*/*z* 369.1336 [M + H]^+^ (calcd. for C_21_H_21_O_6_, 369.1338), *m*/*z* 391.1144 [M + Na]^+^ (calcd. for C_21_H_20_O_6_Na, 391.1158); NMR data (DMSO-*d*_6_), see Table 1; Table 2.

Sinoflavonoid NB (**13**): yellow, amorphous powder; UV (MeOH) λmax (log ε) 260 (0.23), 376 (0.18) nm; IR*ν*_max_ 3419, 2955, 2924, 2852, 1651, 1596, 1556, 1456, 1363, 1323, 1159, 1084 cm^−1^; HR-ESI-MS (positive): *m*/*z* 371.0989 [M + H]^+^ (calcd. for C_20_H_19_O_7_, 371.1131), (negative): *m*/*z* 369.0972 [M − H]^−^ (calcd. for C_20_H_17_O_7_, 369.0974); NMR data (DMSO-*d*_6_), see Table 1; Table 2.

Sinoflavonoid NC (**14**): yellow, amorphous powder; [α${]}_{D}^{25}$–9.1 (c 0.07, MeOH); UV (MeOH) λmax (log ε) 264 (0.15), 342 (0.06) nm; IR*ν*_max_ 3279, 2975, 2925, 2854, 1654, 1588, 1487, 1452, 1356, 1260, 1159, 1117, 1088 cm^−1^; HR-ESI-MS (positive): *m*/*z* 469.1853 [M + H]^+^ (calcd. for C_26_H_29_O_8_, 469.1862); NMR data (DMSO-*d*_6_), see Table 1; Table 2.

Sinoflavonoid ND (**15**): yellow, amorphous powder; [α${]}_{D}^{25}$–13.0 (c 0.08, MeOH); UV (MeOH) λmax (log ε) 264 (0.15), 342 (0.06) nm; IR*ν*_max_ 3387, 2950, 2878, 2832, 1654, 1599, 1540, 1490, 1377, 1315, 1255, 1137, 1106, 1070 cm^−1^; HR-ESI-MS (positive): *m*/*z* 499.1966 [M + H]^+^ (calcd. for C_27_H_31_O_9_, 499.1968), *m*/*z* 521.1792 [M + Na]^+^ (calcd. for C_27_H_30_O_9_Na, 521.1788); NMR data (DMSO-*d*_6_), see Table 1; Table 2.

Sinoflavonoid NE (**16**): yellow, amorphous powder; [α${]}_{D}^{25}$–12.8 (c 0.11, MeOH); UV (MeOH) λmax (log ε) 265 (0.57), 349 (0.25) nm; IR*ν*_max_ 3364, 2974, 2931, 1651, 1591, 1485, 1447, 1359, 1226, 1147 cm^−1^; HR-ESI-MS (positive): *m*/*z* 469.1860 [M + H]^+^ (calcd. for C_26_H_29_O_8_, 469.1862); NMR data (DMSO-*d*_6_), see Table 1; Table 2.

### 3.5. Cytotoxicity Asssay

Tumor cells were maintained in RPMI-1640 medium containing 10% heat-inactivated fetal bovine serum, penicillin (100 units/mL), streptomycin (100 ug/mL) under humidified air with 5% CO_2_ at 37 °C. Exponentially growing cells were seeded into 96-well tissue culture-treated plates and precultured for 24h. The isolates were tested against MCF-7 and HepG2 cell lines, using an established MTT assay protocol [11].

## 4. Conclusions

Prenylated flavonoids are characterized by the presence of lipophilic prenylated group on the parent skeleton. Their structure diversity is most attributed to the different position of prenylation, and various length, further cyclization and hydroxylation of prenyl chain. With diverse chemical structure, prenylated flavonoids exhibit extensive pharmacological actions, including antioxidant, anti-inflammatory, anticoagulant, antiviral, antimicrobial anticancer [22], antigenotoxic [23], antiplasmodial [24] and estrogen regulation activities [25]. However, currently, 80% of the approximately 1100 prenylated flavonoids exist in only three plant families (Asteraceae, Cannabinaceae, Leguminosae) [22]. Consequently, their exploitation and use is limited by the narrow distribution in the plant kingdom. Forty-six flavonoids, including 37 prenylated ones, were isolated from *S. hexandrum* [2,8,9,10,11]. Most of them were tested for the cytotoxic activity in tumor cell lines [2,9,11]. Further phytochemical studies on *S. hexandrum* resulted in the isolation of 16 new prenylated flavonoids and eight known analogues. Their cytotoxic activity was evaluated against MCF-7 and HepG2 cell lines. Compound **6** was the most valuable of all tested compounds. Further research is necessary to elucidate the antitumor mechanism. This study also enriches the chemical and pharmacological diversity of prenylated flavonoids.

## Figures and Tables

**Table 1:** ^1^H-NMR Spectroscopic Data (500 MHz, DMSO-*d*_6_) of **1**–**16**.

**No.**	**1**	**2**	**3**	**4**	**5**	**6**	**7**	**8**
6	6.27 s						6.31 s	6.29 s
8		6.35 s	6.28 s	6.34 s	6.40 s	6.53 s		
2′					7.46 d (2.2)	7.54 d (2.2)		
5′	6.70 d (8.2)	6.76 d (8.3)	6.68 d (8.2)	6.73 d (8.4)	6.86 d (8.5)	6.90 d (8.5)	6.90 d (8.4)	6.79 d (8.4)
6′	6.74 d (8.2)	6.74 d (8.3)	6.72 d (8.2)	6.76 d (8.4)	7.35 dd (8.5, 2.2)	7.43 dd (8.5, 2.2)	7.59 d (8.4)	7.09 d (8.4)
1″	3.21 d (6.8)	3.21 d (7.1)	2.54 d (6.8)	2.62 t (6.7)	2.54 t (6.7)	3.06 d (8.4)	3.30 d (6.8)	3.30 d (6.8)
2″	5.01 t (6.8)	5.16 t (7.1)	1.74 t (6.8)	1.81 t (6.7)	1.72 t (6.7)	4.75 t (8.4)	5.06 t (6.8)	5.05 t (6.8)
4″	1.45 s	1.61 s	1.30 s	1.30 s	1.30 s	1.13 s	1.49 s	1.58 s
5″	1.53 s	1.71 s	1.30 s	1.30 s	1.30 s	1.14 s	1.56 s	1.60 s
1‴	3.25 d (6.8)	3.24 d (7.3)	3.21 d (6.9)	3.25 d (6.9)			7.05 d (2.1)	3.44 dd (17.1, 6.8) 2.95 dd (17.1, 2.4)
2‴	4.94 t (6.8)	5.03 t (7.3)	5.04 t (6.9)	5.00 t (6.9)			8.09 d (2.1)	6.03 br.s
4‴	1.27 s	1.34 s	1.34 s	1.30 s				
5‴	1.39 s	1.49 s	1.50 s	1.45 s				
OCH_3_		3.56 s	3.48 s	3.56 s	3.69 s	3.77 s	3.65 s	3.64 s
5-OH	12.46 s	12.90 s		13.00 s		12.92 s	12.70 s	12.65 s
7-OH	10.64 s	10.81 s	10.60 s		10.67 s		10.84 s	
**No.**	**9**	**10**	**11**	**12**	**13**	**14**	**15**	**16**
6	6.17 s	6.30 s	6.30 s	6.18 s	6.12 s	6.17 s	6.16 s	6.30 s
8				6.43 s				
2′				7.80 d (2.0)	7.73 d (2.1)			
5′	6.77 s	6.75 d (8.2)	6.90 d (8.4)	6.87 d (8.5)	6.89 d (8.5)	6.75 d (8.5)	6.76 d (8.2)	6.75 d (8.2)
6′	6.77 s	6.87 d (8.2)	7.55 d (8.4)	7.78 d (8.5, 2.0)	7.60 dd (8.5, 2.1)	6.92 d (8.5)	6.89 d (8.2)	6.90 d (8.2)
1″	2.79 dd (16.5, 5.2) 2.47 dd (16.5, 7.1)	3.23 d (6.9)	2.67 m	2.83 d (6.5)	2.84 t (6.6)	2.83 dd (16.5, 5.1) 2.54 dd (16.5, 6.7)	4.20 d (2.7)	3.13 d (8.5)
2″	3.67 dd (7.1, 5.2)	5.05 t (6.9)	1.47 m	1.82 d (6.5)	1.87 t (6.6)	3.69 dd (6.7, 5.1)	3.80 m	4.74 t (8.5)
4″	1.20 s	1.48 s	0.98 s	1.32 s	1.32 s	1.23 s	1.32 s	1.13s
5″	1.27 s	1.54 s	0.98 s	1.32 s	1.32 s	1.27 s	1.36 s	1.14 s
1‴	3.23 d (6.9)	2.75 dd (17.0, 5.4) 2.55 dd (17.0, 8.2)	7.01 s			2.68 t (6.6)	2.63 m	2.66 m
2‴	4.99 t (6.9)	3.60dd (8.2, 5.4)				1.73 t (6.6)	1.71 m	1.73 m
4‴	1.27 s	1.17 s	5.77 s 5.28 s			1.32 s	1.31 s	1.30 s
5‴	1.44 s	1.32 s	2.10 s			1.32 s	1.31 s	1.31 s
OCH_3_	3.57 s	3.57 s	3.65 s	3.78 s		3.58 s	3.59 s	3.57 s
OCH_3_							3.31 s	
5-OH	12.45 s	12.57 s	12.67 s	12.65 s	12.24 s	12.44 s	12.65 s	12.87 s
7-OH		10.81 s						

**Table 2:** ^13^C-NMR Spectroscopic Data (100 MHz, DMSO-*d*_6_) of **1**–**16**.

**No.**	**1**	**2**	**3**	**4**	**5**	**6**	**7**	**8**
2	150.7 s	159.6 s	155.1 s	159.7 s	151.4 s	155.7 s	156.6 s	156.0 s
3	136.2 s	139.0 s	140.6 s	138.5 s	139.7 s	137.7 s	137.7 s	138.0 s
4	176.5 s	178.5 s	172.0 s	178.2 s	172.1 s	178.0 s	178.1 s	178.1 s
5	158.3 s	158.5 s	154.6 s	158.2 s	154.5 s	155.3 s	160.0 s	159.0 s
6	97.7 d	111.1 s	105.0 s	104.4 s	105.0 s	108.8 s	98.2 d	98.2 d
7	160.8 s	162.3 s	159.8 s	159.8 s	159.8 s	166.2 s	161.6 s	161.6 s
8	105.4 s	93.3 d	93.2 d	94.4 d	93.1 d	88.5 d	106.0 s	105.9 s
9	154.2 s	154.9 s	156.4 s	154.4 s	156.0 s	156.2 s	153.9 s	153.9 s
10	103.5 s	104.7 s	107.6 s	104.3 s	107.2 s	105.1 s	104.3 s	104.4 s
1′	123.0 s	123.2 s	121.8 s	121.1 s	121.4 s	120.7 s	113.4 s	117.8 s
2′	128.1 s	128.2 s	127.6 s	127.8 s	115.0 d	115.7 d	128.1 s	127.0 s
3′	143.0 s	143.7 s	143.2 s	143.3 s	145.1 s	145.2 s	143.1 s	145.4 s
4′	146.5 s	147.4 s	146.5 s	147.1 s	147.7 s	148.1 s	145.5 s	143.3 s
5′	112.4 d	112.9 d	112.4 d	112.4 d	115.6 d	115.4 d	110.4 d	115.2 d
6′	121.0 d	121.5 d	120.9 d	121.0 d	119.8 d	120.5 d	125.9 d	122.3 d
1″	20.9 t	21.4 t	16.7 t	15.7 t	16.7 t	25.7 t	21.1 t	21.1 t
2″	121.99 d	122.6 d	30.8 t	30.9 t	30.8 t	91.5 d	122.4 d	122.4 d
3″	130.6 s	131.1 s	74.7 s	76.2 s	74.8 s	70.0 s	131.0 s	131.3 s
4″	17.3 q	18.1 q	26.4 q	26.3 q	26.4 q	24.8 q	17.6 q	17.7 q
5″	25.4 q	25.9 q	26.4 q	26.3 q	26.4 q	25.9 q	25.3 q	25.4 q
1‴	25.7 t	26.2 t	26.0 t	25.7 q			107.3 d	38.2 t
2‴	122.04 d	123.3 d	122.9 d	122.8 d			146.6 d	101.0 d
3‴	129.8 s	130.6 s	129.9 s	130.2 s				
4‴	17.2 q	17.8 q	17.4 q	17.4 q				
5‴	25.2 q	25.7 q	25.4 q	25.4 q				
OCH_3_		60.3 q	59.4 q	59.9 q	59.2 q	59.6 q	60.6 q	60.2 q
**No.**	**9**	**10**	**11**	**12**	**13**	**14**	**15**	**16**
2	158.7 s	158.2 s	156.7 s	156.6 s	147.7 s	158.3 s	158.0 s	158.0 s
3	139.1 s	138.7 s	137.7 s	138.2 s	136.1 s	139.2 s	139.5 s	138.8 s
4	178.2 s	178.2 s	178.9 s	178.3 s	175.9 s	178.1 s	178.1 s	178.1 s
5	159.4 s	159.0 s	158.7 s	161.6 s	158.0 s	158.75 s	160.3 s	161.8 s
6	98.9 d	98.2 d	98.2 d	99.0 d	98.5 d	98.8 d	98.7 d	93.3 d
7	158.7 s	161.5 s	161.7 s	164.7 s	159.1 s	158.82 s	158.8 s	166.2 s
8	99.0 s	105.9 s	107.2 s	94.1 d	99.7 s	99.0 s	100.3 s	104.4 s
9	154.1 s	154.1 s	154.1 s	155.8 s	153.0 s	154.0 s	156.0 s	151.3 s
10	105.4 s	104.5 s	104.4 s	104.6 s	103.6 s	105.5 s	105.8 s	105.4 s
1′	121.2 s	120.5 s	113.5 s	121.7 s	122.2 s	120.3 s	120.2 s	120.2 s
2′	127.7 s	120.6 s	129.4 s	130.2 d	114.8 d	121.3 s	121.0 s	121.1 s
3′	143.3 s	141.0 s	142.7 s	121.5 s	145.1 s	142.0 s	141.9 s	142.0 s
4′	147.1 s	147.9 s	144.9 s	156.8 s	146.7 s	148.3 s	148.2 s	148.3 s
5′	112.5 d	112.9 d	111.0 d	117.5 d	115.7 d	112.3 d	112.8 d	112.7 d
6′	121.0 d	121.5 d	125.9 d	128.1 d	120.2 d	121.1 d	121.4 d	121.2 d
1″	24.9 t	21.0 t	17.5 t	22.2 t	15.7 t	24.7 t	73.9 d	25.2 t
2″	66.8 d	122.0 d	47.8 t	32.5 t	30.9 t	66.6 d	67.3 d	91.5 d
3″	78.9 s	130.9 s	68.7 s	75.8 s	76.2 s	78.9 s	78.8 s	69.9 s
4″	20.8 q	17.4 q	28.8 q	27.0 q	26.3 q	20.2 q	23.1 q	24.8 q
5″	25.1 q	25.5 q	28.8 q	27.0 q	26.3 q	25.2 q	24.5 q	25.7 q
1‴	25.7 t	29.5 t	104.2 d			21.1 t	19.9 t	20.1 t
2‴	122.9 d	67.8 d	156.5 s			31.8 t	31.8 t	31.7t
3‴	130.2 s	76.8 s	132.5 s			73.9 s	73.5 s	73.9 s
4‴	17.2 q	19.8 q	114.1 t			26.0 q	26.4 q	26.2 q
5‴	25.2 q	25.3 q	18.9 q			26.0 q	26.4 q	26.6 q
OCH_3_	59.8 q	60.2 q	60.1 q	60.1 q	59.2 q	60.2 q	60.2 q	60.2 q
OCH_3_							56.9 q	

**Table 3:** Cytotoxicities of **1**–**18**, **20**, **22** against MCF-7 and HepG2 cell lines (IC_50_, μM).

Compound	MCF-7	HepG2	Compound	MCF-7	HepG2
**1**	33.8 ± 2.0	75.9 ± 5.3	**15**	25.5 ± 1.8	17.2 ± 1.3
**2**–**5**, **7**–**12**, **17**, **18**	>100	>100	**16**	41.8 ± 3.5	55.4 ± 4.9
**6**	6.25 ± 0.49	3.83 ± 0.26	**20**	48.3 ± 3.2	50.6 ± 4.4
**13**	59.7 ± 4.1	45.3 ± 3.5	**22**	59.3 ± 5.7	>100
**14**	30.4 ± 2.6	23.1 ± 1.7	**5-fluorouracil**	33.4 ± 3.0	18.2 ± 2.5

## Supplementary Materials

The following are available online. Figures S1–S64: NMR spectra of compounds **1**–**16**.
