# Supplementary material for: Sixteen New Prenylated Flavonoids from the Fruit of Sinopodophyllum hexandrum
Source: Molecules. 2019 Sep 3;24(17):3196. doi: 10.3390/molecules24173196 (PMC6749350; doi:10.3390/molecules24173196)
Supplement: Supplementary file 1 [file molecules-24-03196-s001.pdf]

Supporting Information

Sixteen New Prenylated Flavonoids from the Fruit of *Sinopodophyllum hexandrum*

Yanjun Sun [1,2,*], Haojie Chen[1,2], Junmin Wang [1,2], Meiling Gao[1,2], Chen Zhao[1,2], Ruijie Han[1,2], Hui Chen [1,2], Meng Li [1,2], Guimin Xue[1,2] and Weisheng Feng [1,2,*]

[1] Collaborative Innovation Center for Respiratory Disease Diagnosis and Treatment & Chinese Medicine, Development of Henan Province, Henan University of Chinese Medicine, Zhengzhou 450046, China

[2] School of Pharmacy, Henan University of Chinese Medicine, Zhengzhou 450046, China

* Correspondence: sunyanjun2011@hactcm.edu.cn (Y.S.); fwsh@hactcm.edu.cn (W.F.); Tel.: +86-371-6596-2746 (Y.S. & W.F.)

Abstract: Sixteen new prenylated flavonoids, sinoflavonoids P–Z (**1–11**) and sinoflavonoids NA–NE (**12–16**), were isolated from the fruit of *Sinopodophyllum hexandrum*, along with eight known analogues (**17–24**). Their structures were elucidated on the basis of extensive spectroscopic data (HR-ESI-MS, ^{1}H-NMR, ^{13}C-NMR, HSQC, HMBC). The cytotoxic activities of compounds **1–18**, **20**, and **22** were evaluated by MTT assay. Compound **6** showed the most potent cytotoxicity in MCF-7, and HepG2 cell lines, with IC_{50} values of 6.25 and 3.83 μM, respectively.

Keywords: *Sinopodophyllum hexandrum*; prenylated flavonoid; cytotoxic activity

Content

The 1D and 2D NMR spectra of compound **1** ... 3
The 1D and 2D NMR spectra of compound **2** ... 5
The 1D and 2D NMR spectra of compound **3** ... 7
The 1D and 2D NMR spectra of compound **4** ... 9
The 1D and 2D NMR spectra of compound **5** ... 11
The 1D and 2D NMR spectra of compound **6** ... 13
The 1D and 2D NMR spectra of compound **7** ... 15
The 1D and 2D NMR spectra of compound **8** ... 17
The 1D and 2D NMR spectra of compound **9** ... 19
The 1D and 2D NMR spectra of compound **10** ... 21
The 1D and 2D NMR spectra of compound **11** ... 23
The 1D and 2D NMR spectra of compound **12** ... 25
The 1D and 2D NMR spectra of compound **13** ... 27
The 1D and 2D NMR spectra of compound **14** ... 29
The 1D and 2D NMR spectra of compound **15** ... 31
The 1D and 2D NMR spectra of compound **16** ... 33

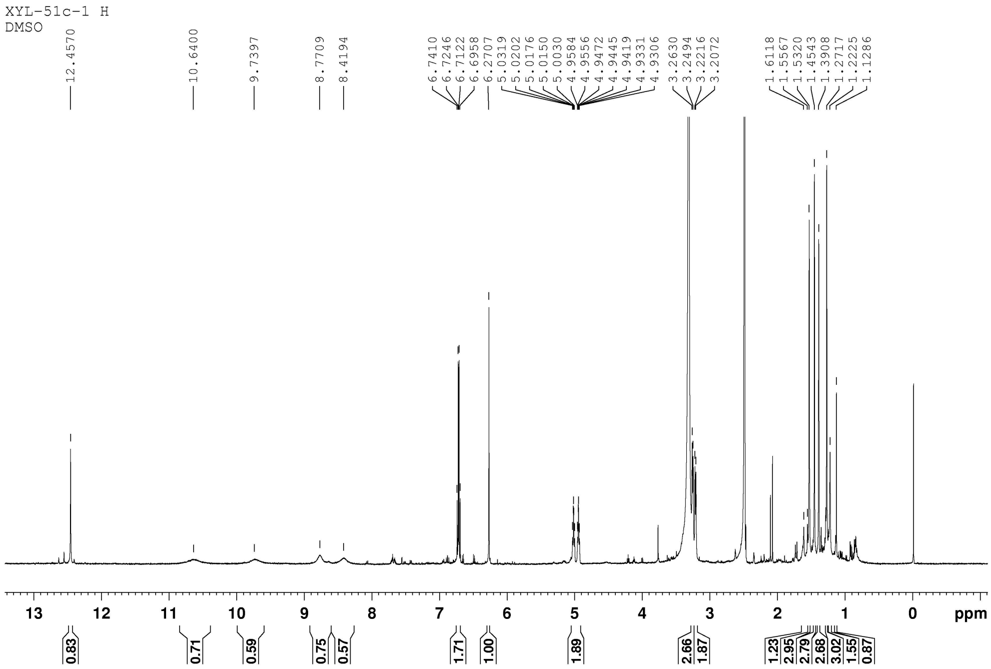

Figure S1. ^{1}H-NMR (500 MHz, DMSO-*d*6) spectrum of compound **1**

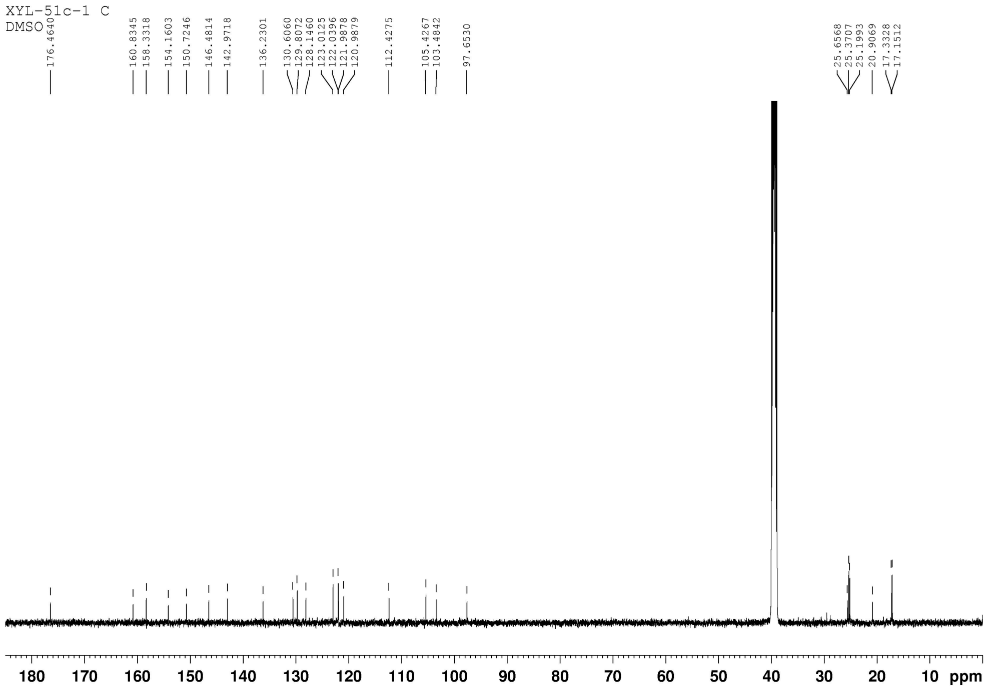

Figure S2. ^{13}C-NMR (125 MHz, DMSO-*d*6) spectrum of compound **1**

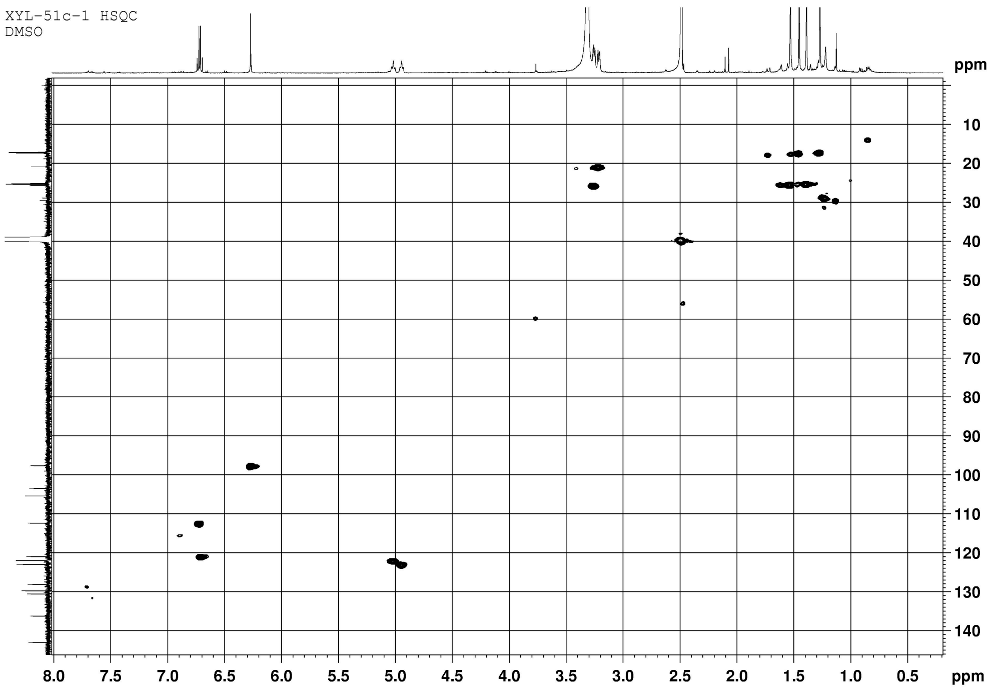

Figure S3. HSQC spectrum of compound **1**

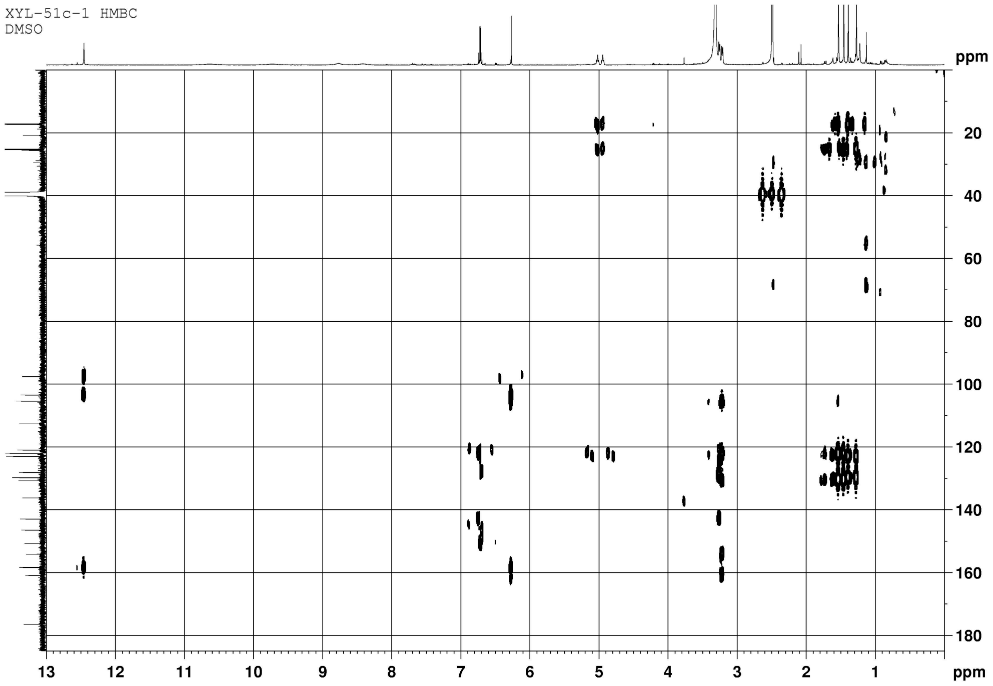

Figure S4. HMBC spectrum of compound **1**

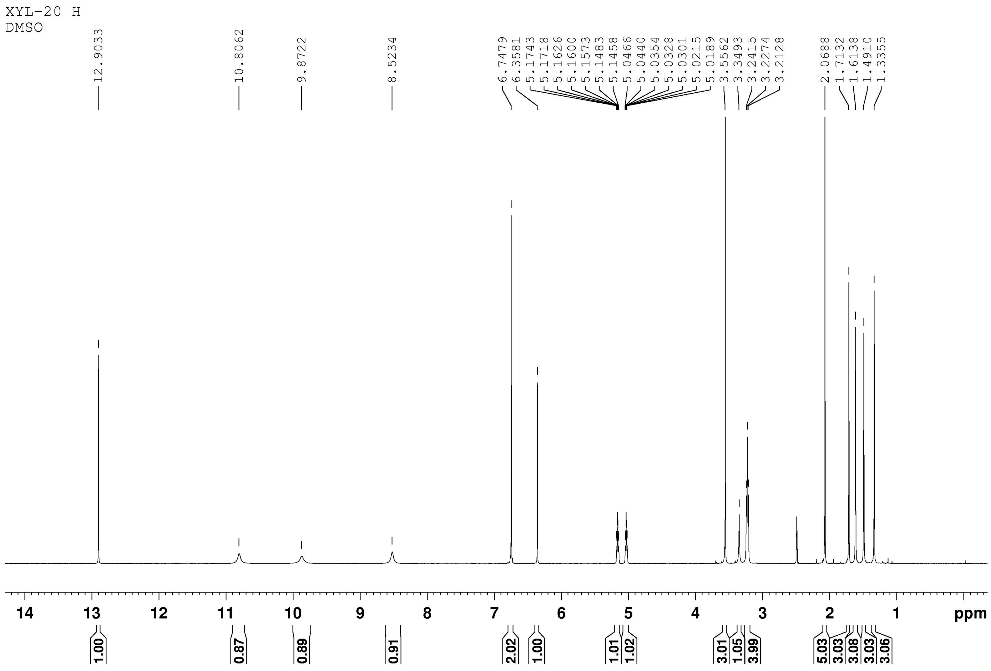

Figure S5. ^{1}H-NMR (500 MHz, DMSO-*d*6) spectrum of compound **2**

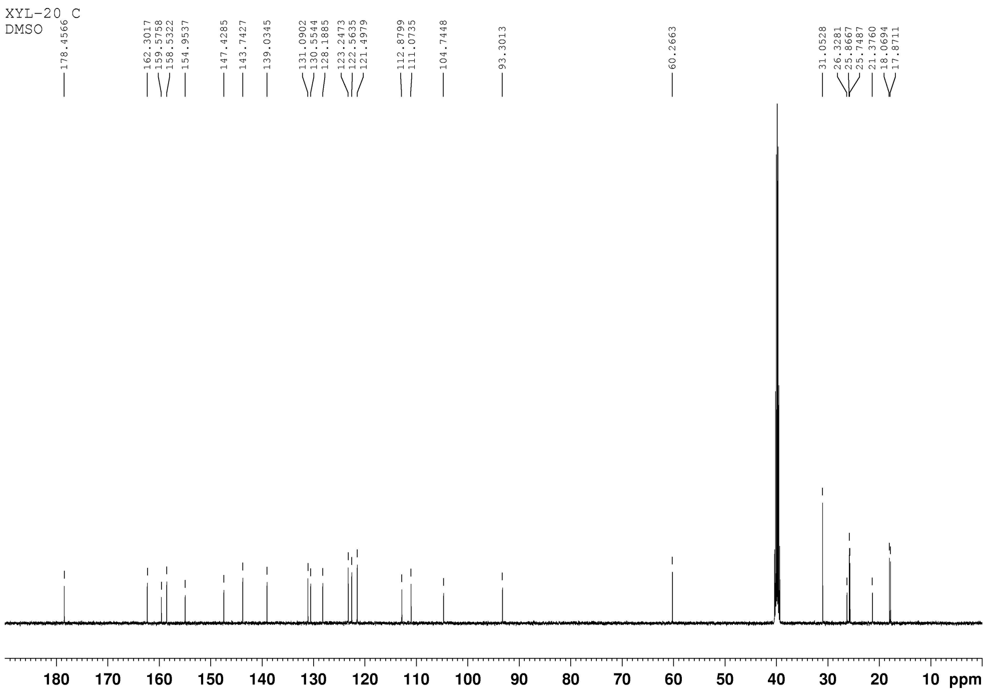

Figure S6. ^{13}C-NMR (125 MHz, DMSO-*d*6) spectrum of compound **2**

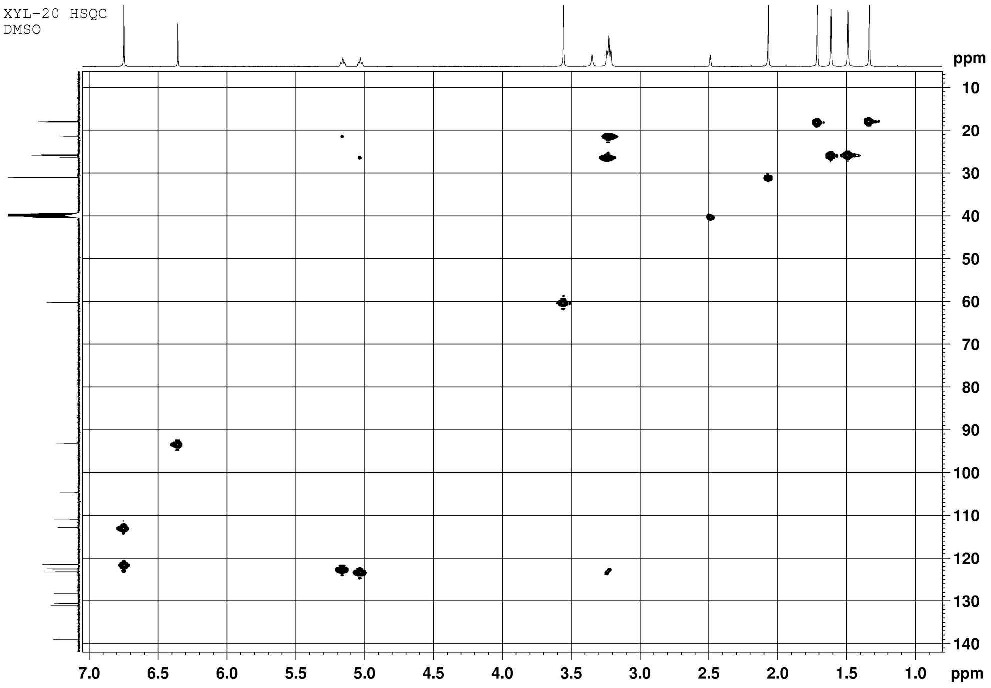

Figure S7. HSQC spectrum of compound **2**

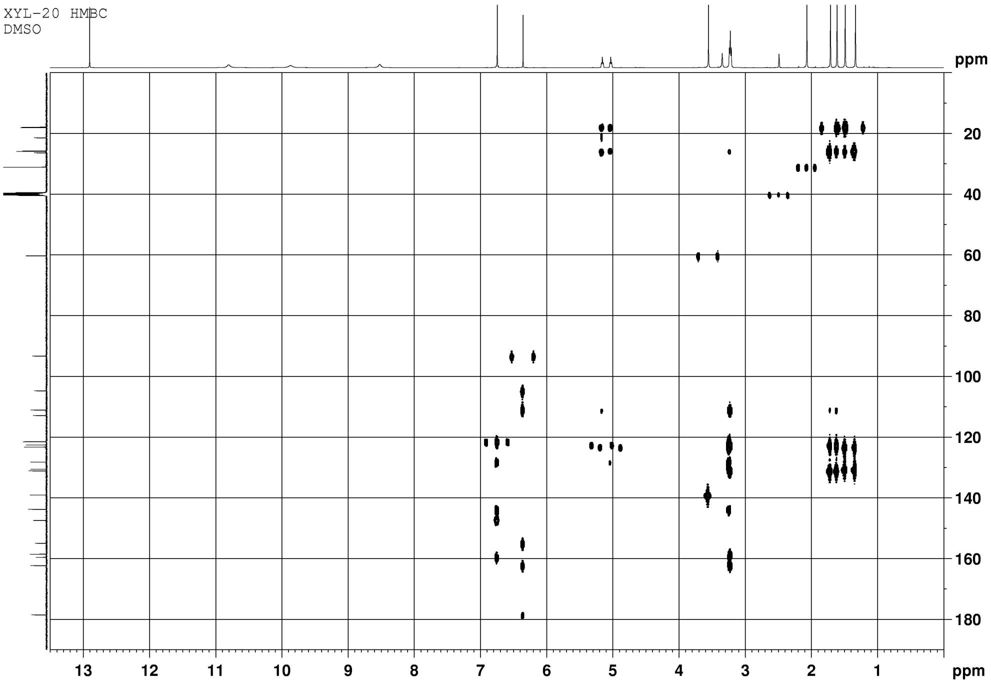

Figure S8. HMBC spectrum of compound **2**

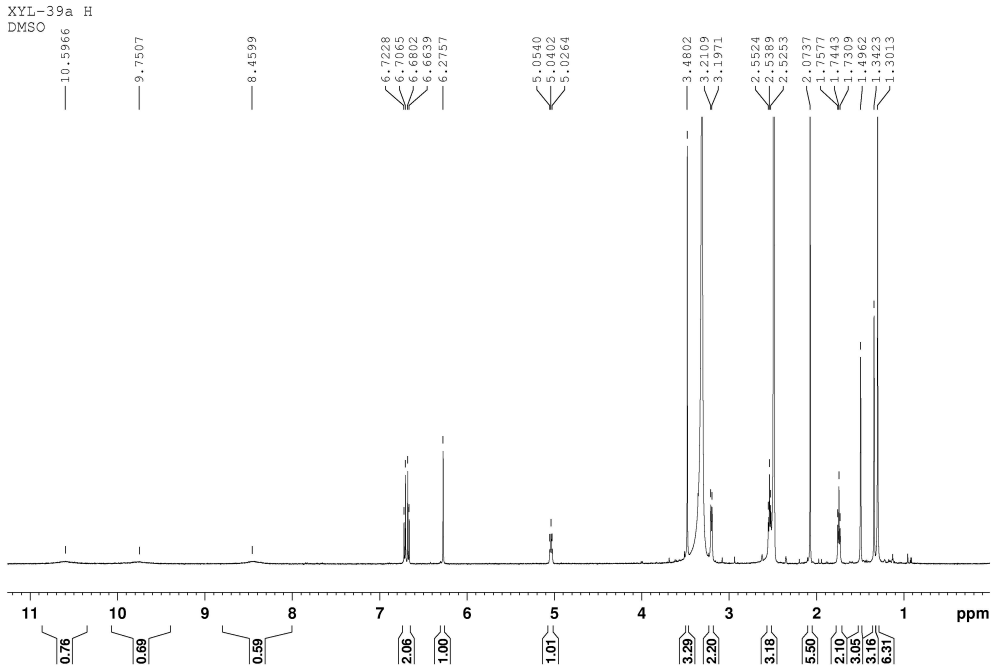

Figure S9. ^{1}H-NMR (500 MHz, DMSO-*d*6) spectrum of compound **3**

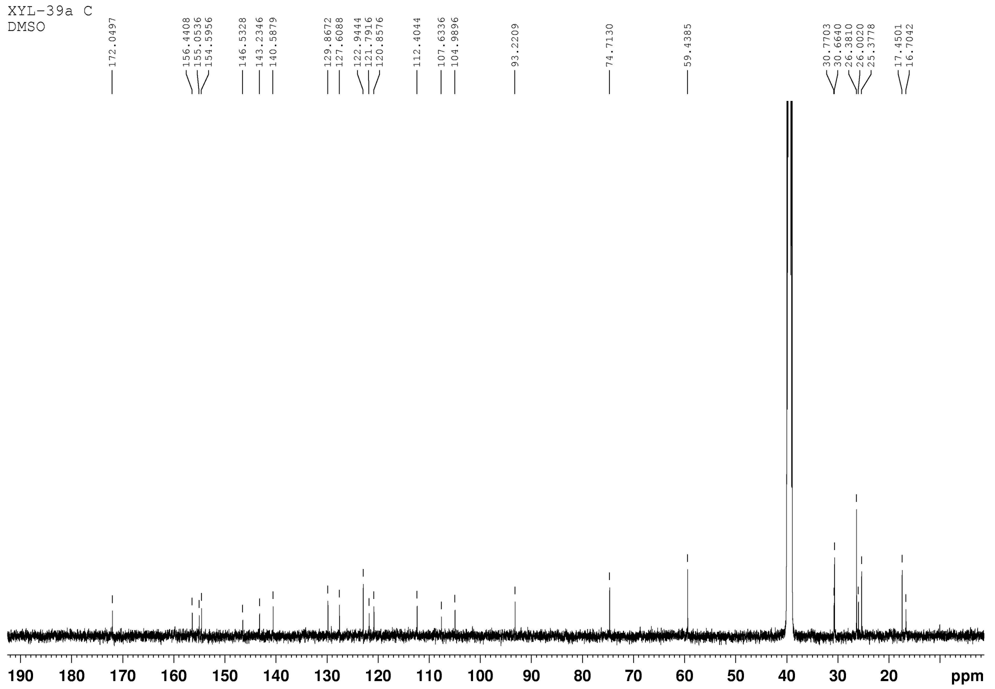

Figure S10. ^{13}C-NMR (125 MHz, DMSO-*d*6) spectrum of compound **3**

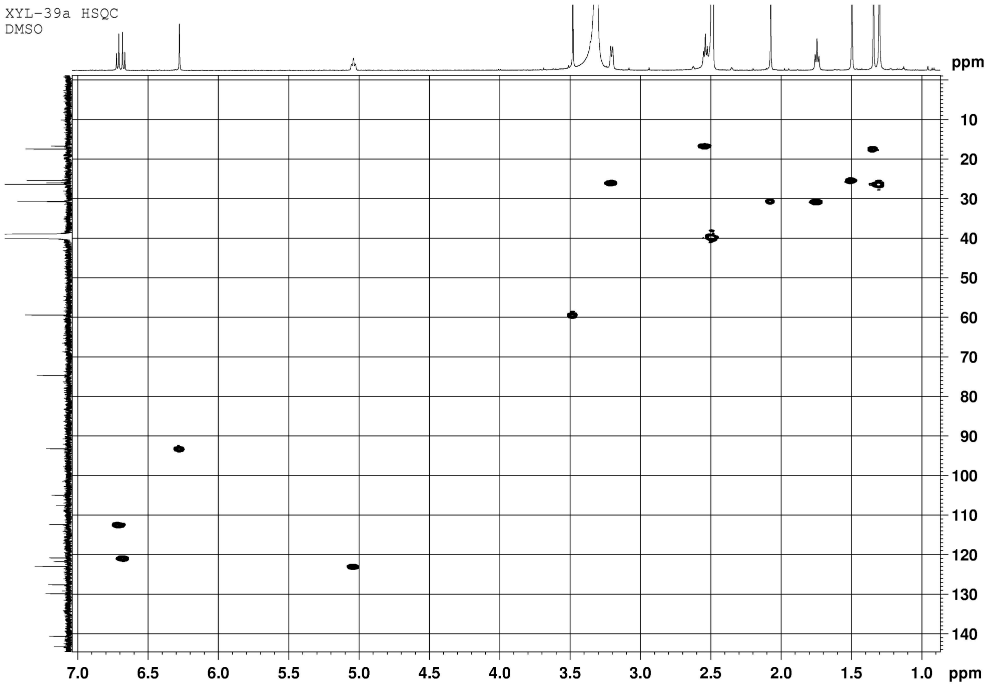

Figure S11. HSQC spectrum of compound **3**

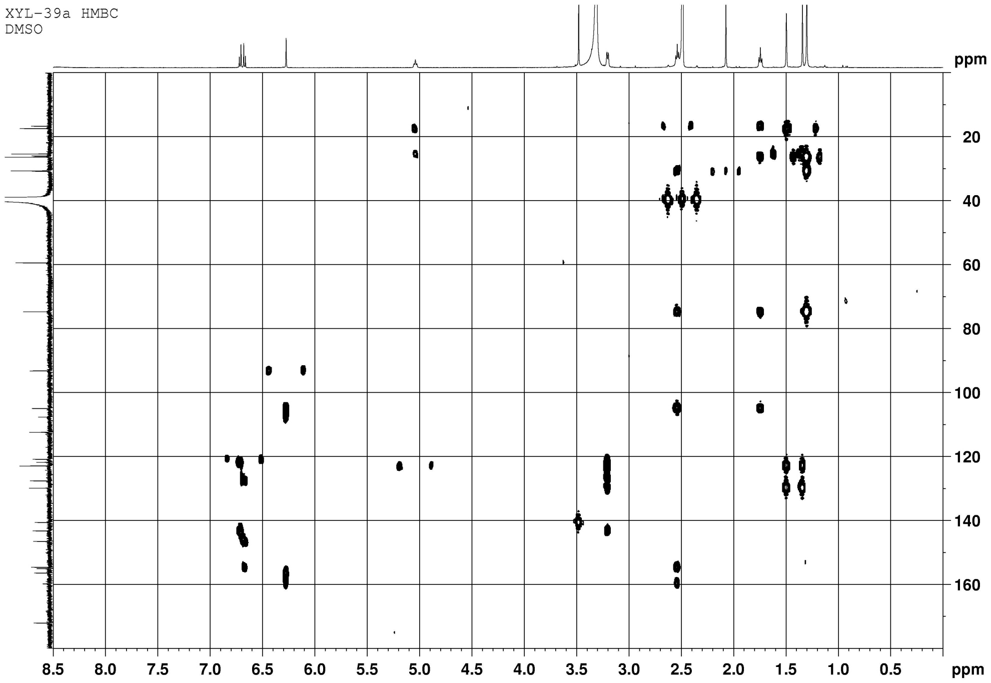

Figure S12. HMBC spectrum of compound **3**

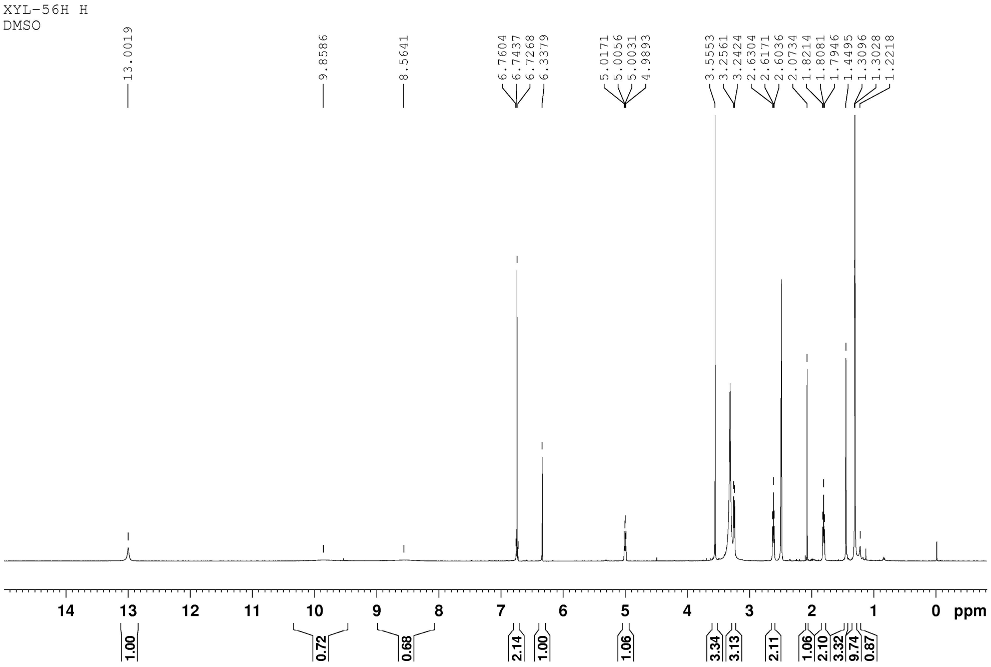

Figure S13. ^{1}H-NMR (500 MHz, DMSO-*d*6) spectrum of compound **4**

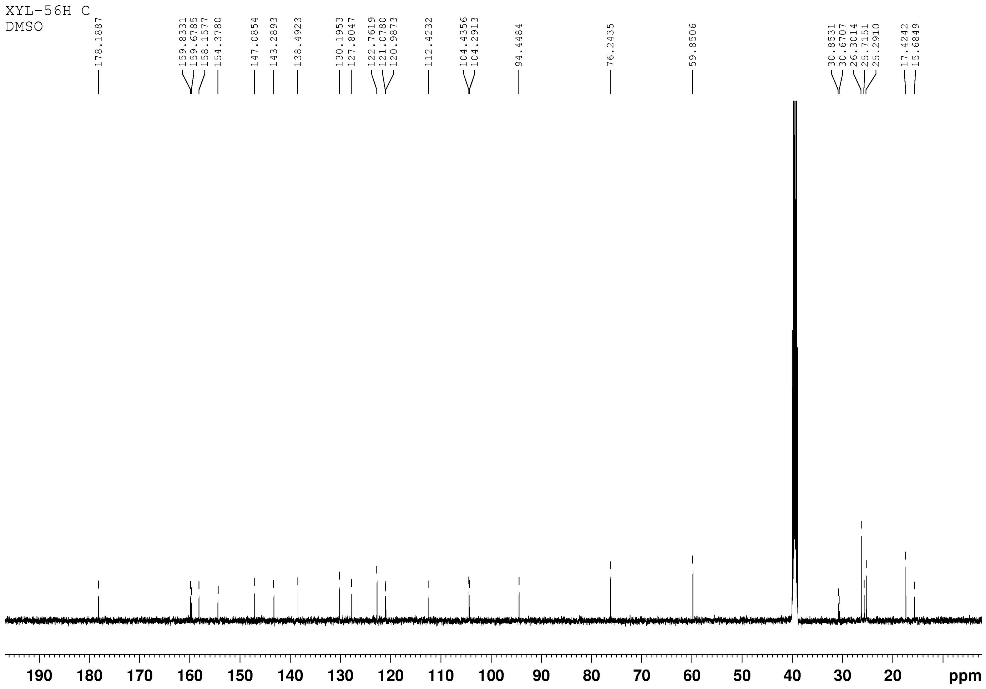

Figure S14. ^{13}C-NMR (125 MHz, DMSO-*d*6) spectrum of compound **4**

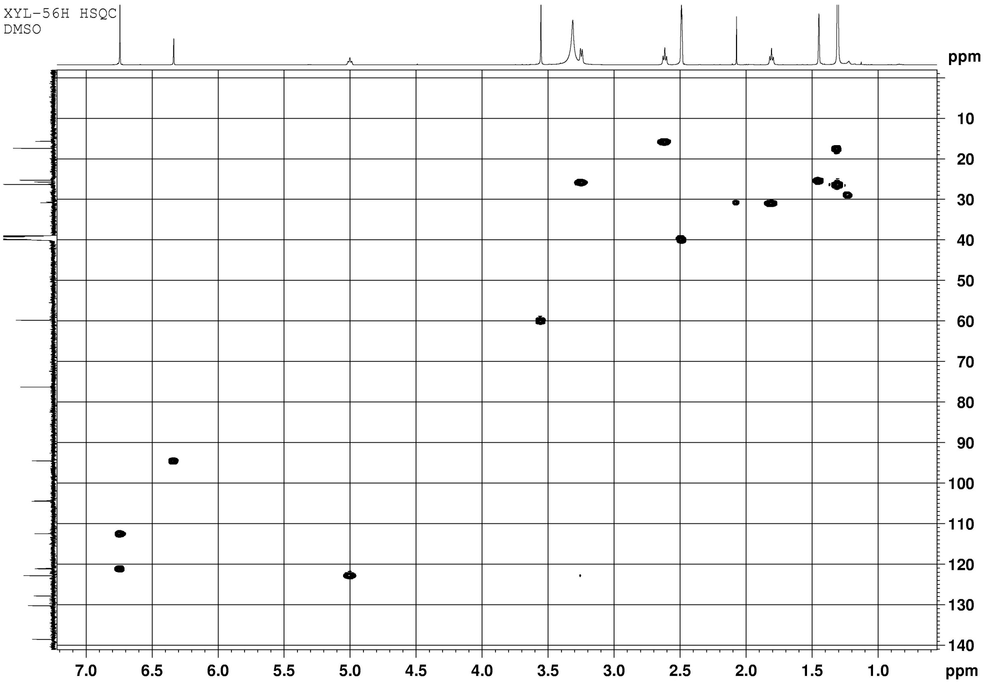

Figure S15. HSQC spectrum of compound **4**

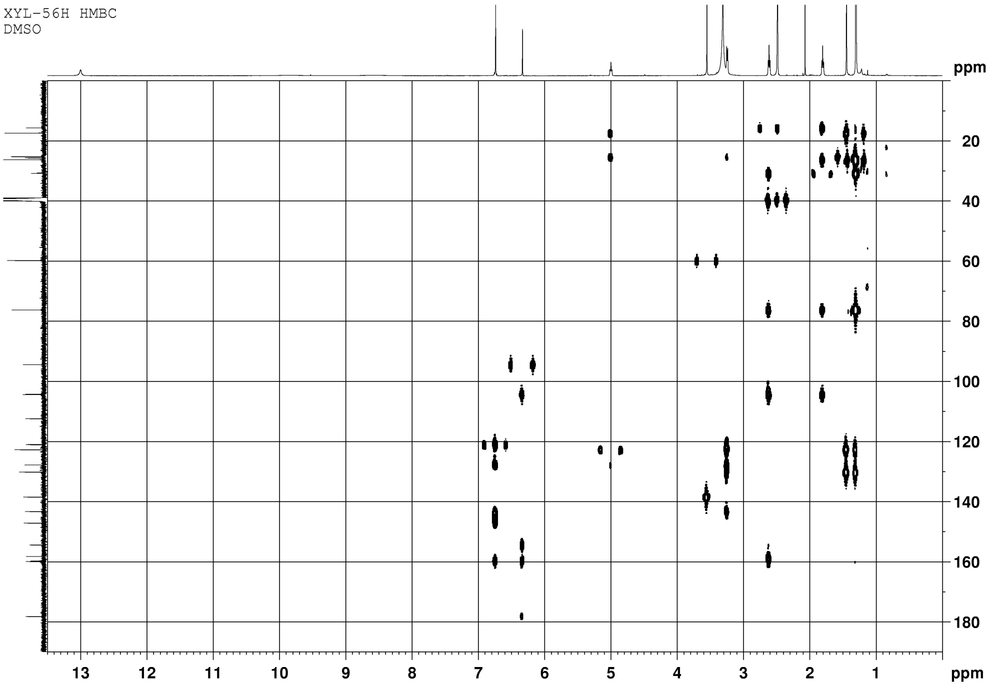

Figure S16. HMBC spectrum of compound **4**

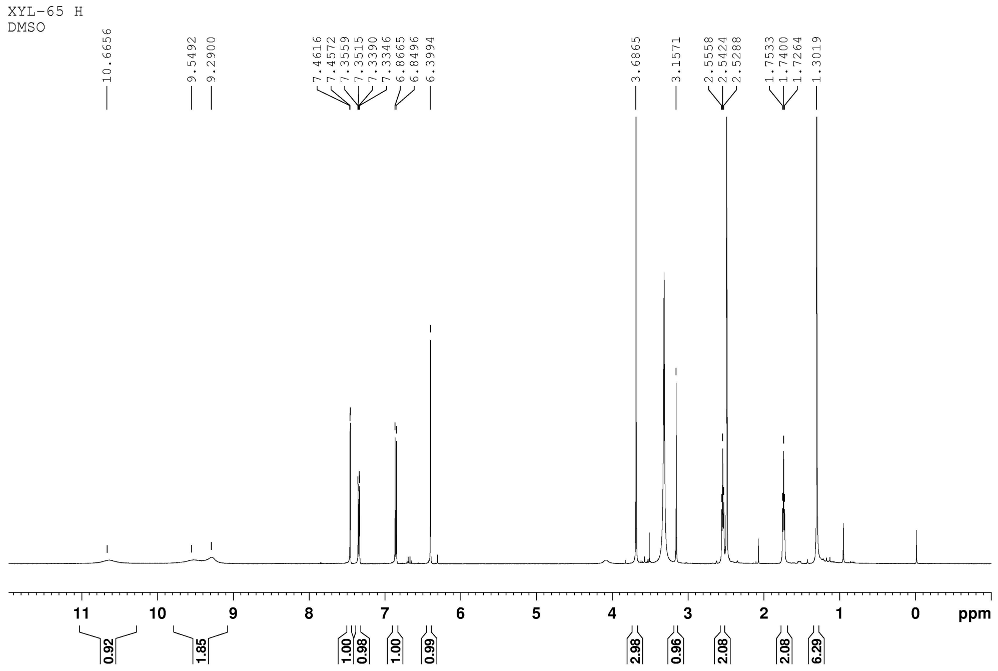

Figure S17. ^{1}H-NMR (500 MHz, DMSO-*d*6) spectrum of compound **5**

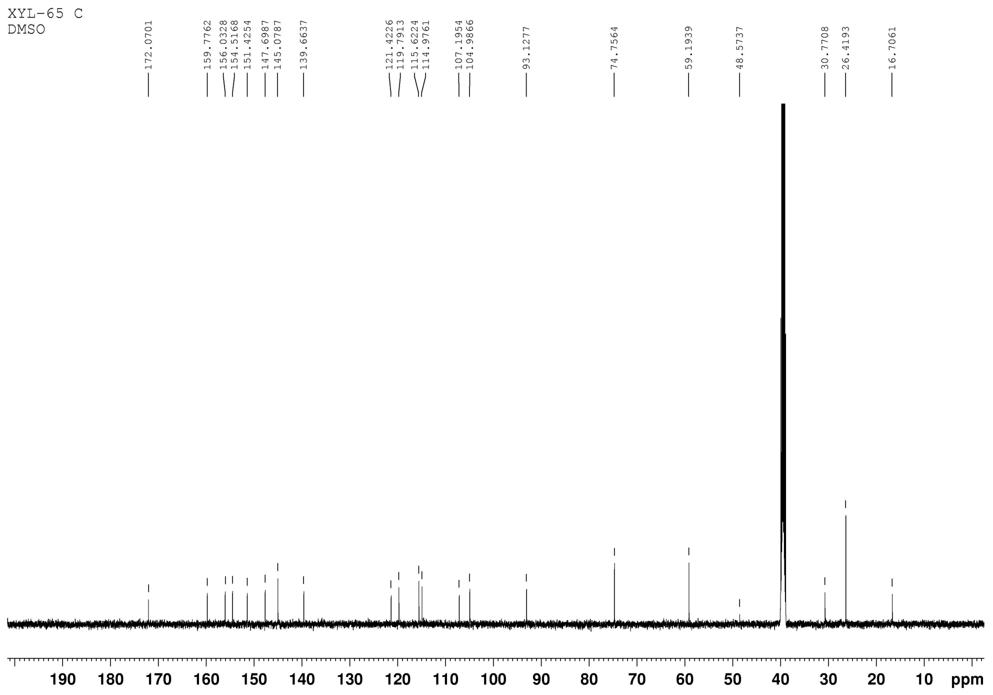

Figure S18. ^{13}C-NMR (125 MHz, DMSO-*d*6) spectrum of compound **5**

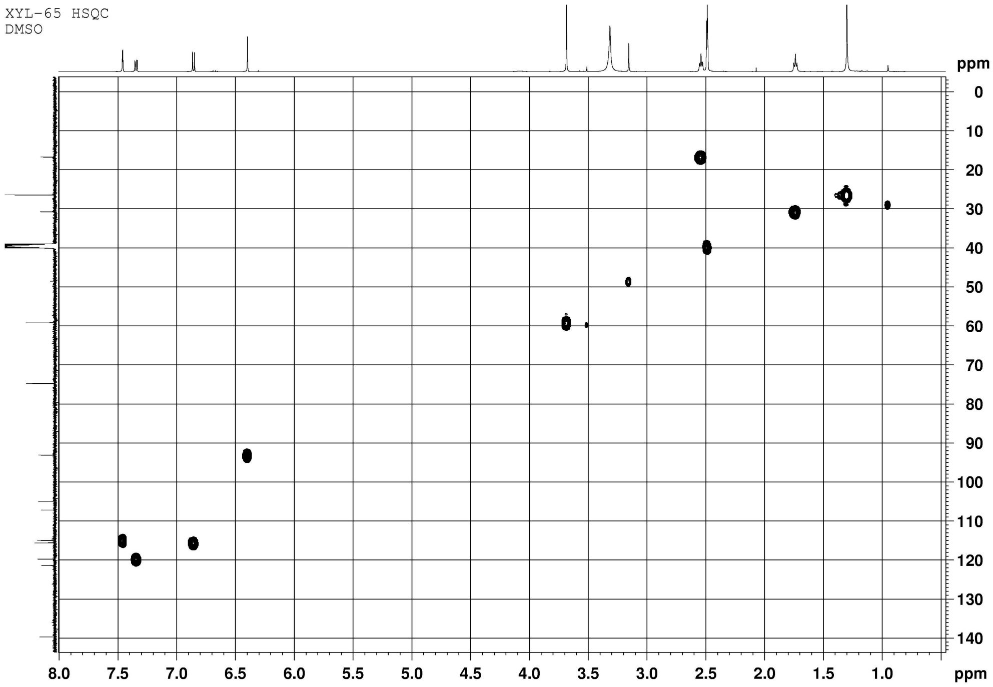

Figure S19. HSQC spectrum of compound **5**

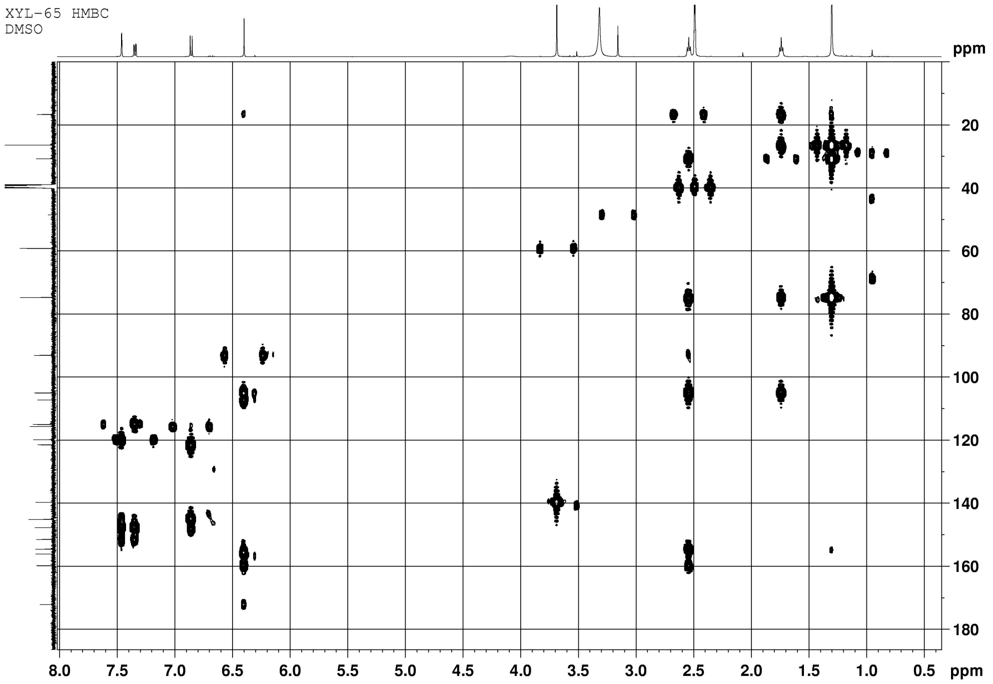

Figure S20. HMBC spectrum of compound **5**

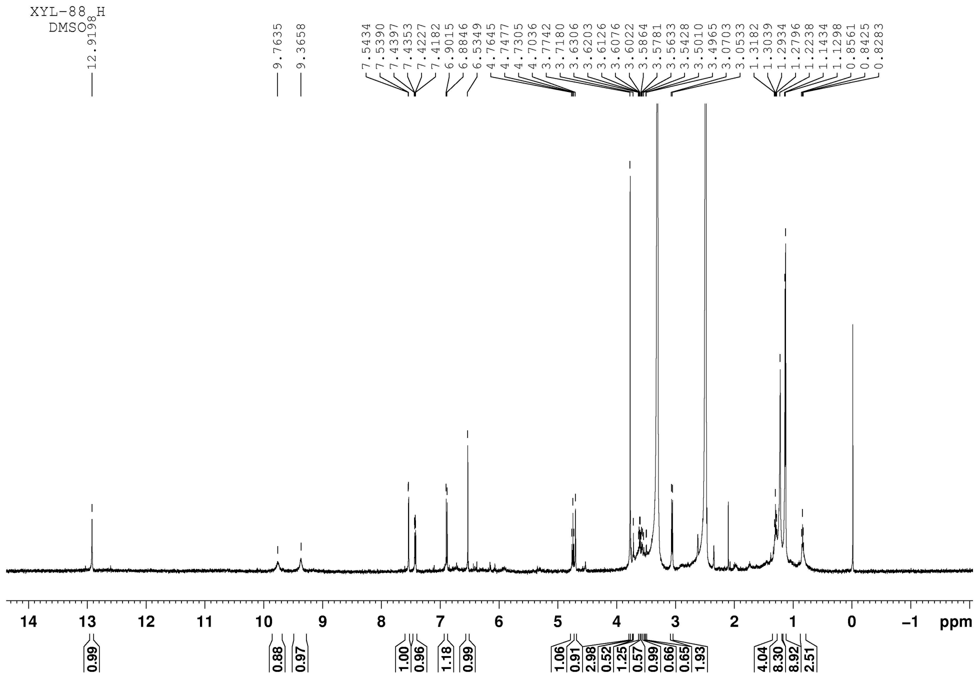

Figure S21. ^{1}H-NMR (500 MHz, DMSO-*d6*) spectrum of compound **6**

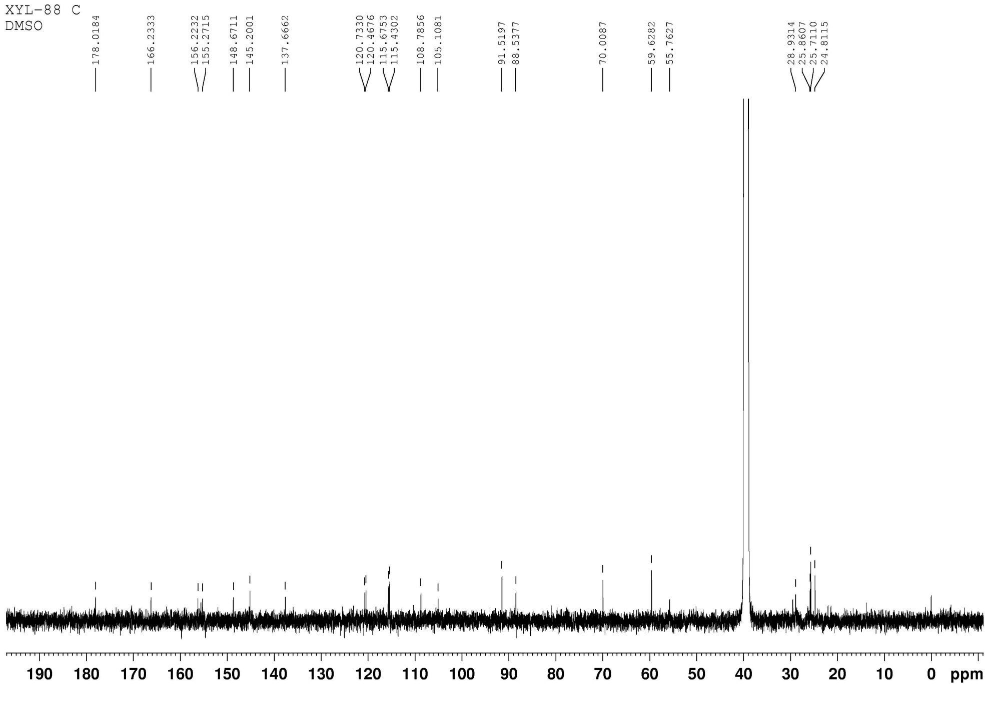

Figure S22. ^{13}C-NMR (125 MHz, DMSO-*d6*) spectrum of compound **6**

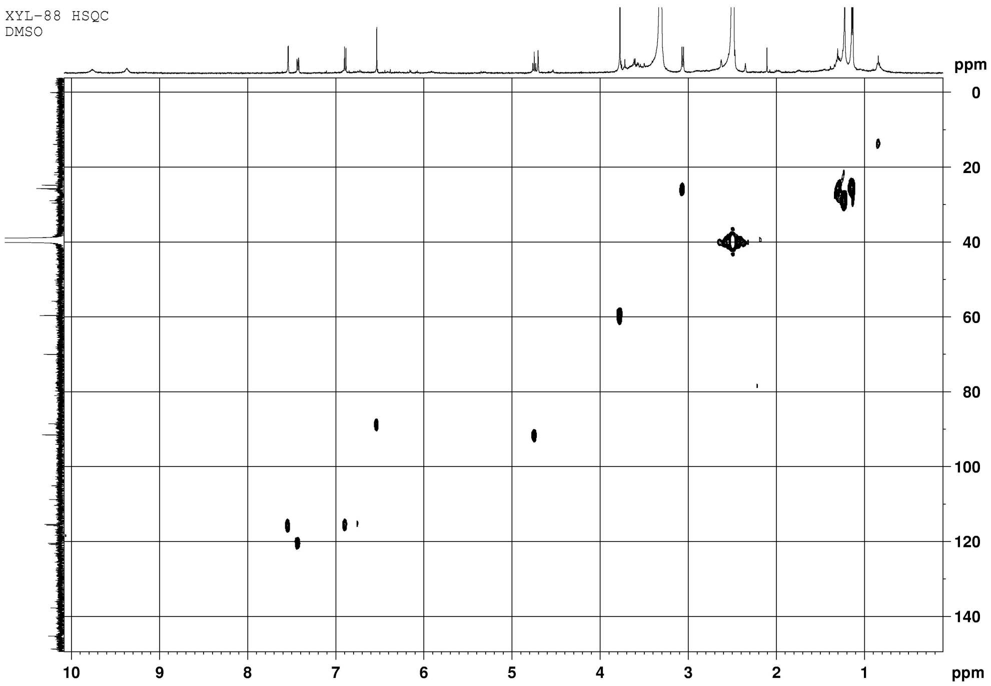

Figure S23. HSQC spectrum of compound **6**

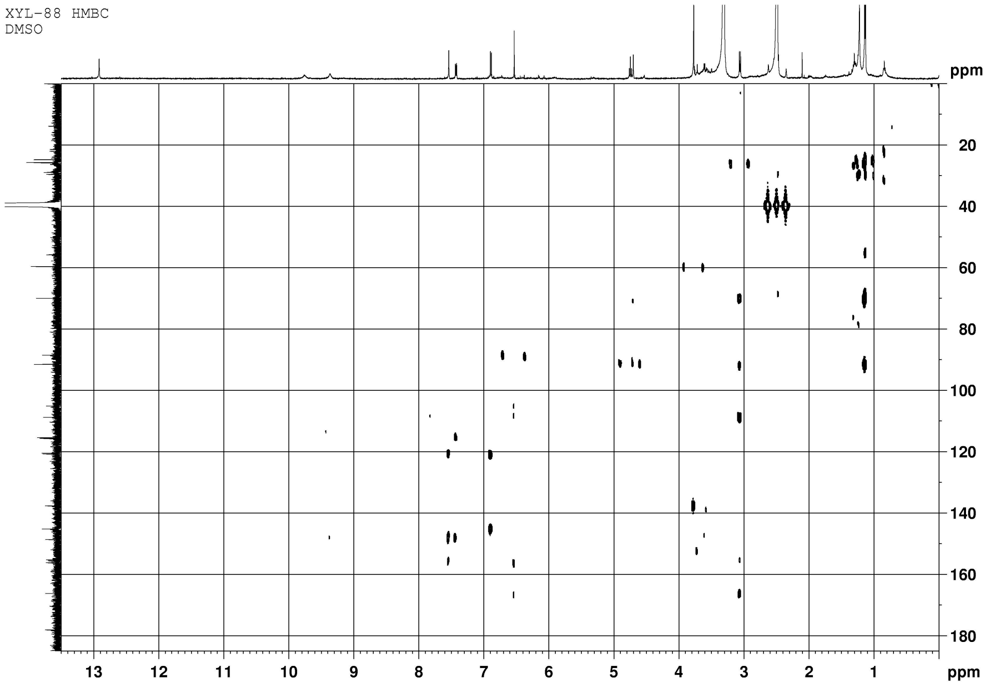

Figure S24. HMBC spectrum of compound **6**

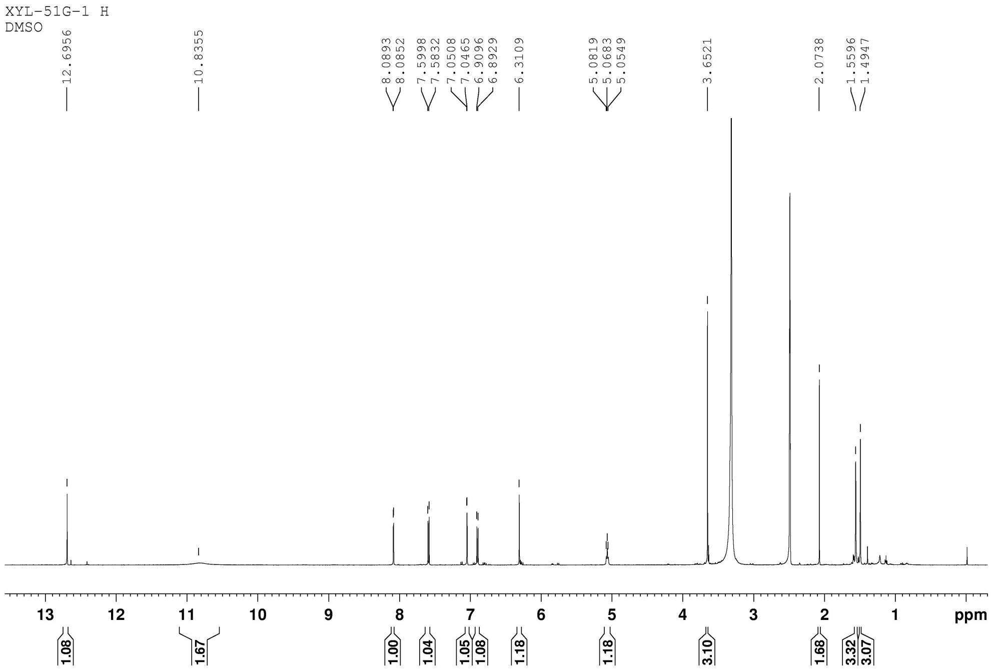

Figure S25. ^{1}H-NMR (500 MHz, DMSO-*d*6) spectrum of compound **7**

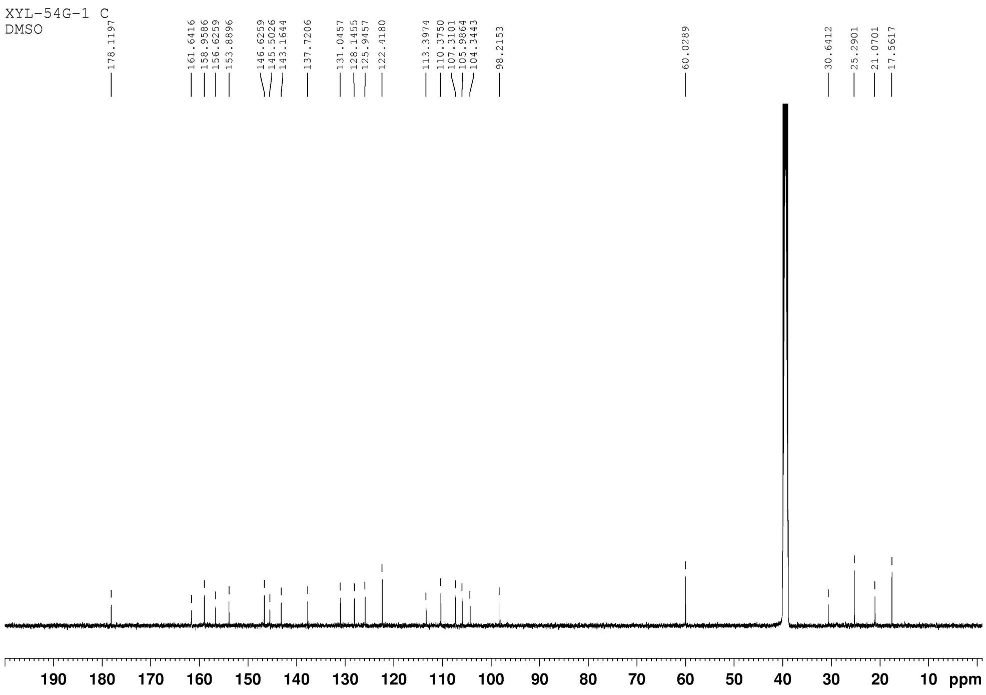

Figure S26. ^{13}C-NMR (125 MHz, DMSO-*d*6) spectrum of compound **7**

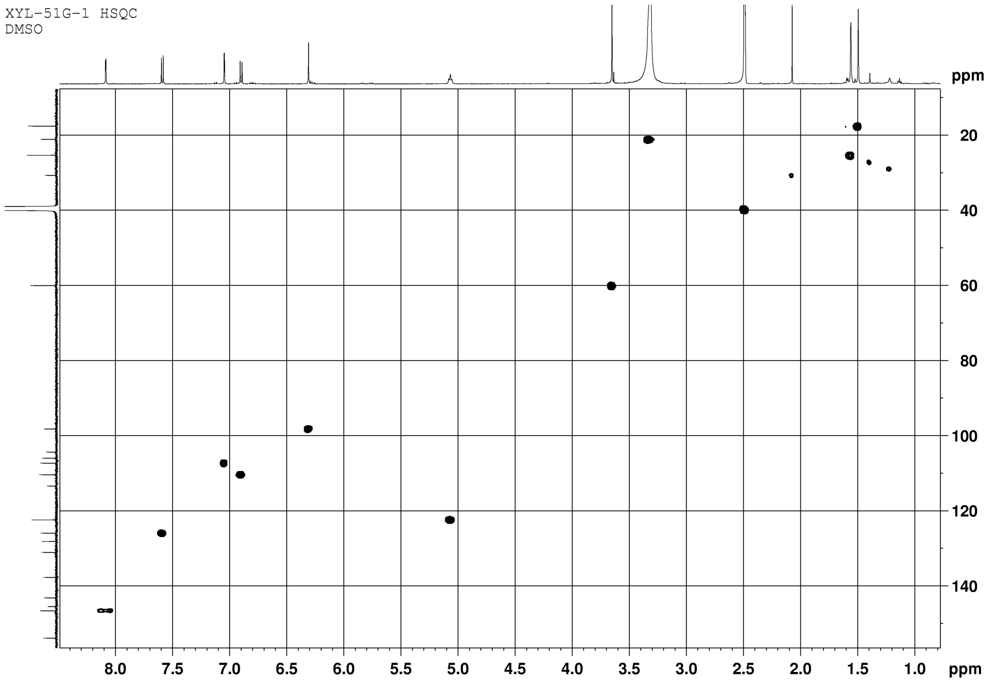

Figure S27. HSQC spectrum of compound **7**

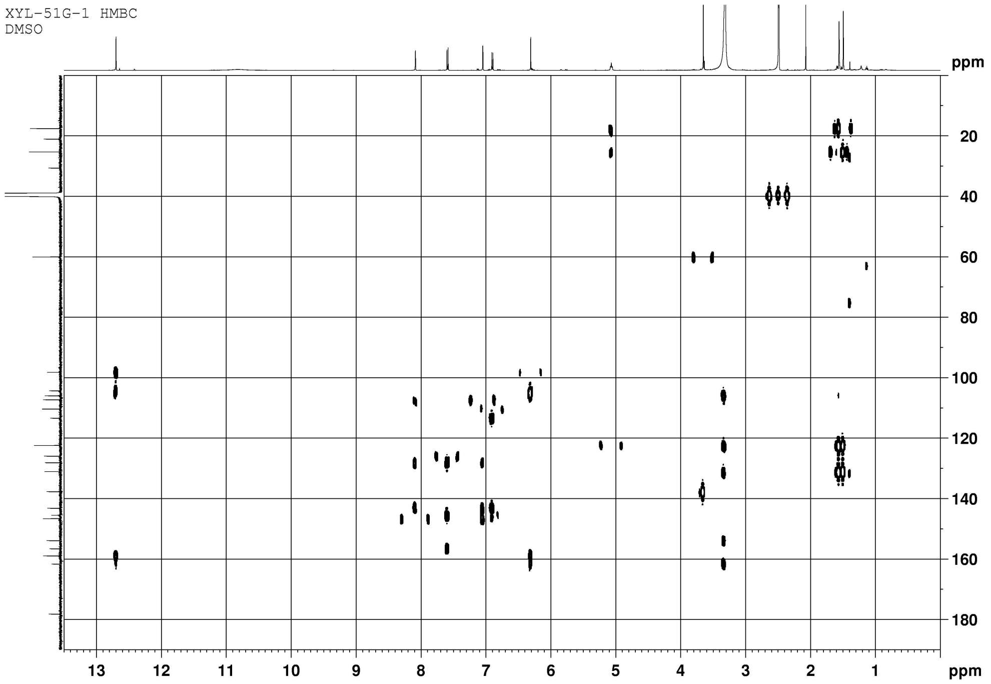

Figure S28. HMBC spectrum of compound **7**

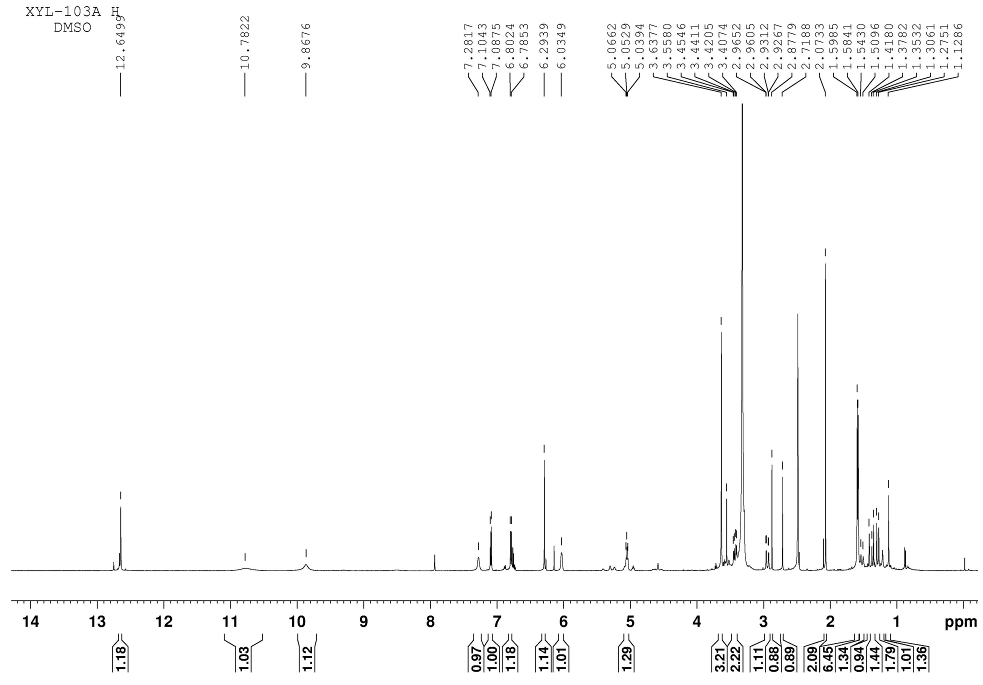

Figure S29. ^{1}H-NMR (500 MHz, DMSO-$d6$) spectrum of compound **8**

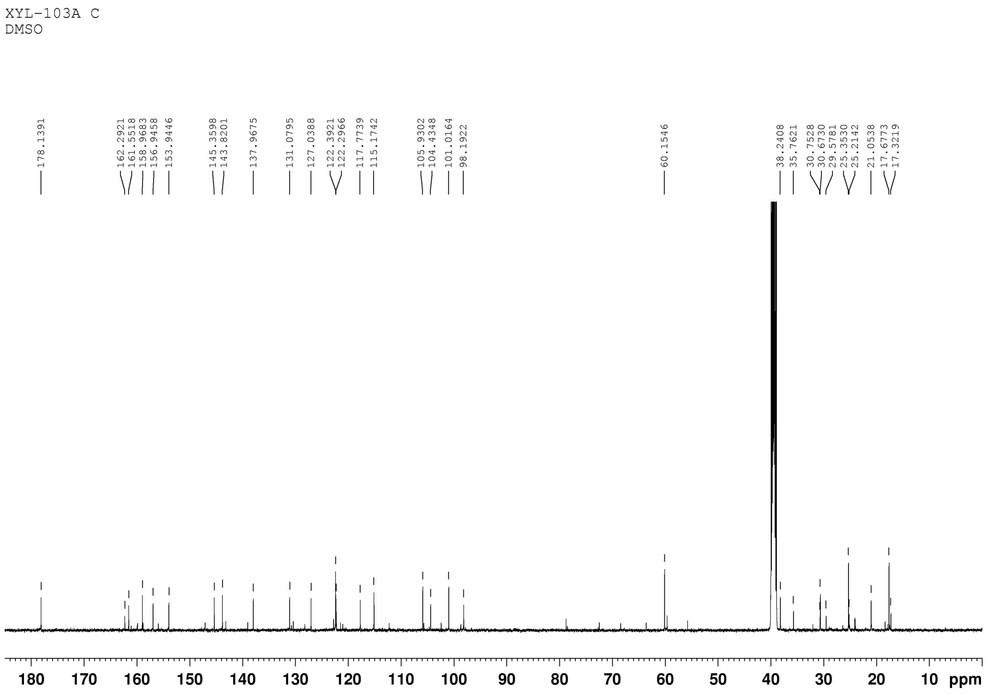

Figure S30. ^{13}C-NMR (125 MHz, DMSO-$d6$) spectrum of compound **8**

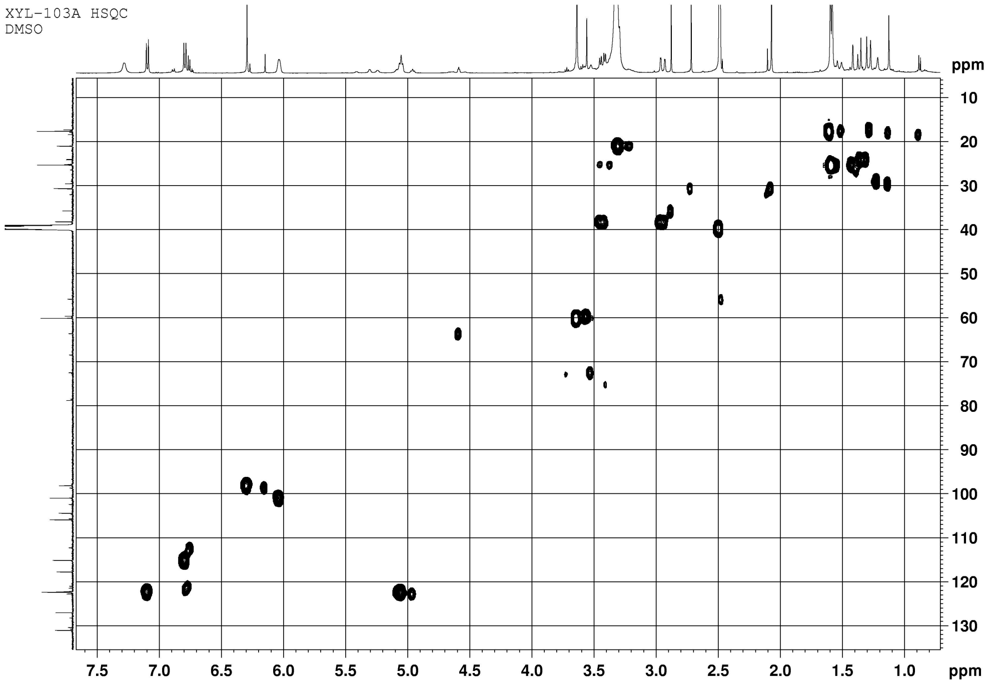

Figure S31. HSQC spectrum of compound **8**

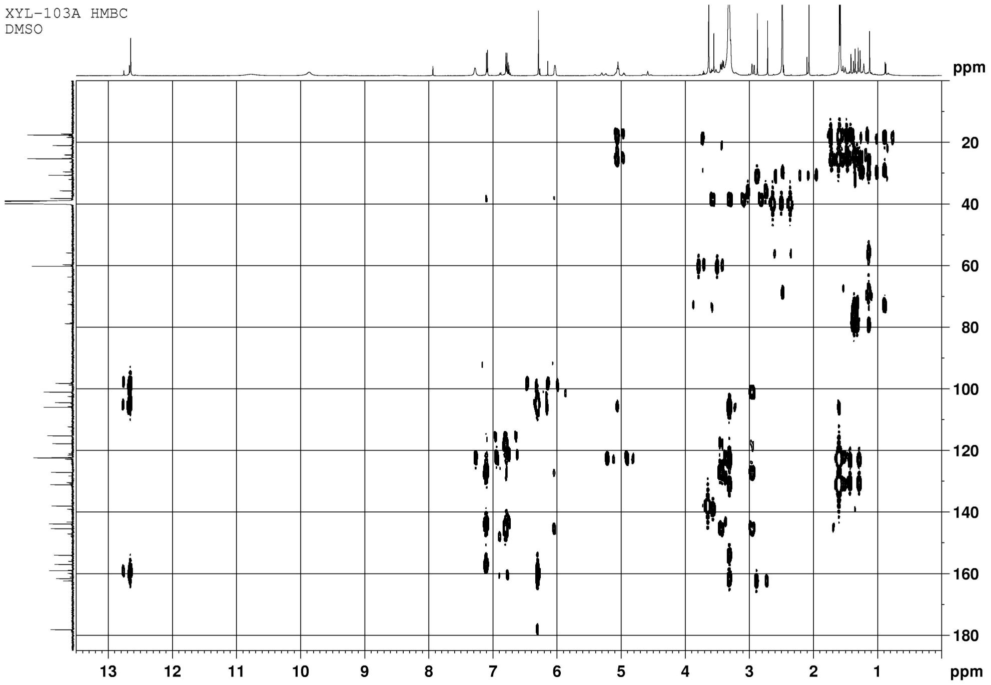

Figure S32. HMBC spectrum of compound **8**

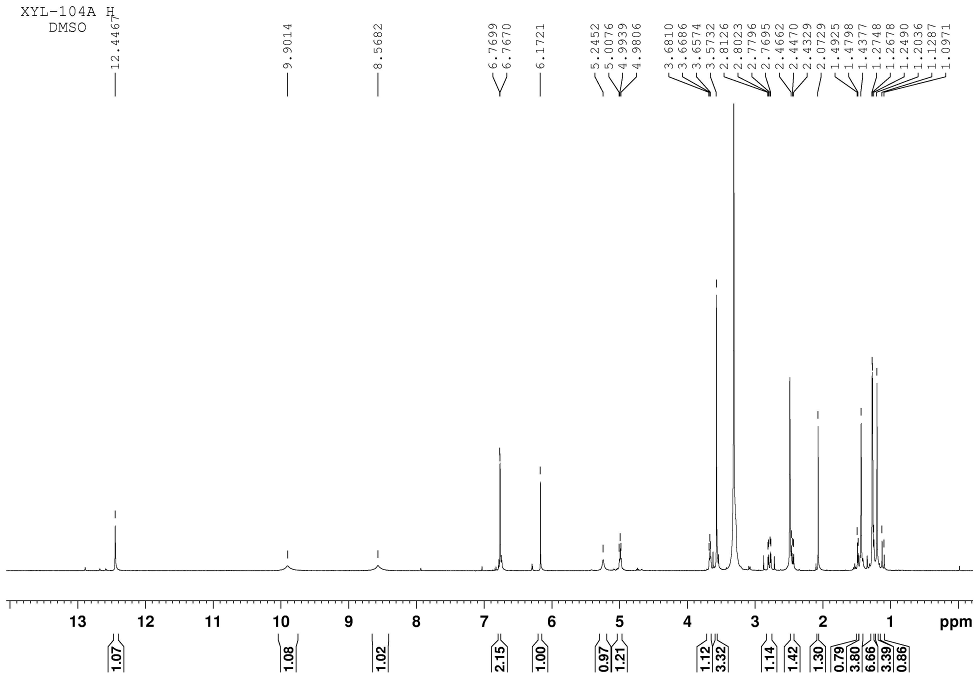

Figure S33. ^{1}H-NMR (500 MHz, DMSO-*d*6) spectrum of compound **9**

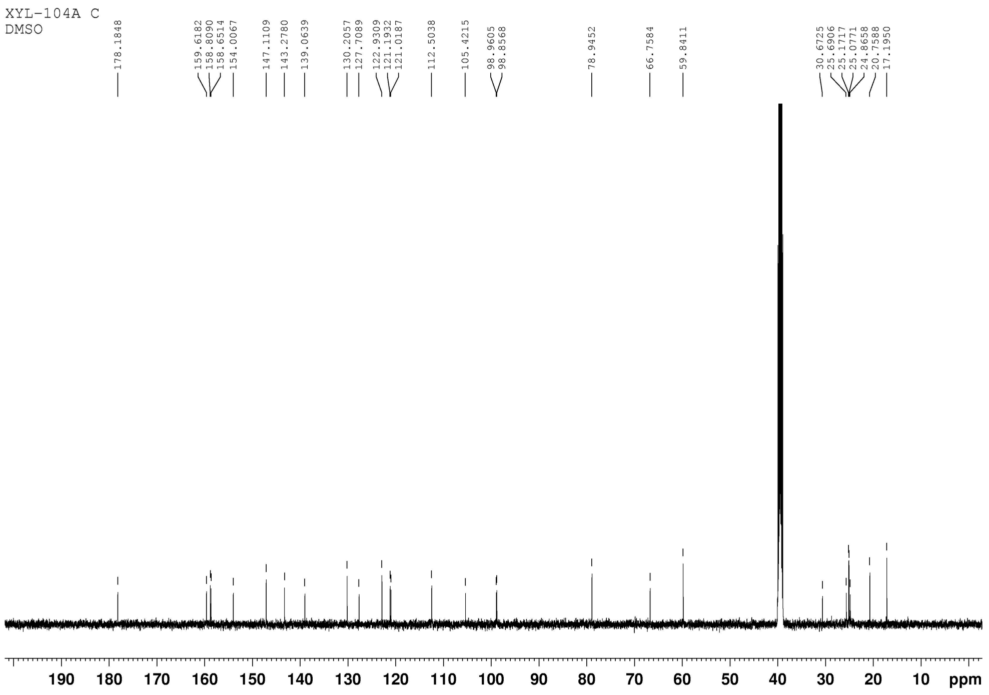

Figure S34. ^{13}C-NMR (125 MHz, DMSO-*d*6) spectrum of compound **9**

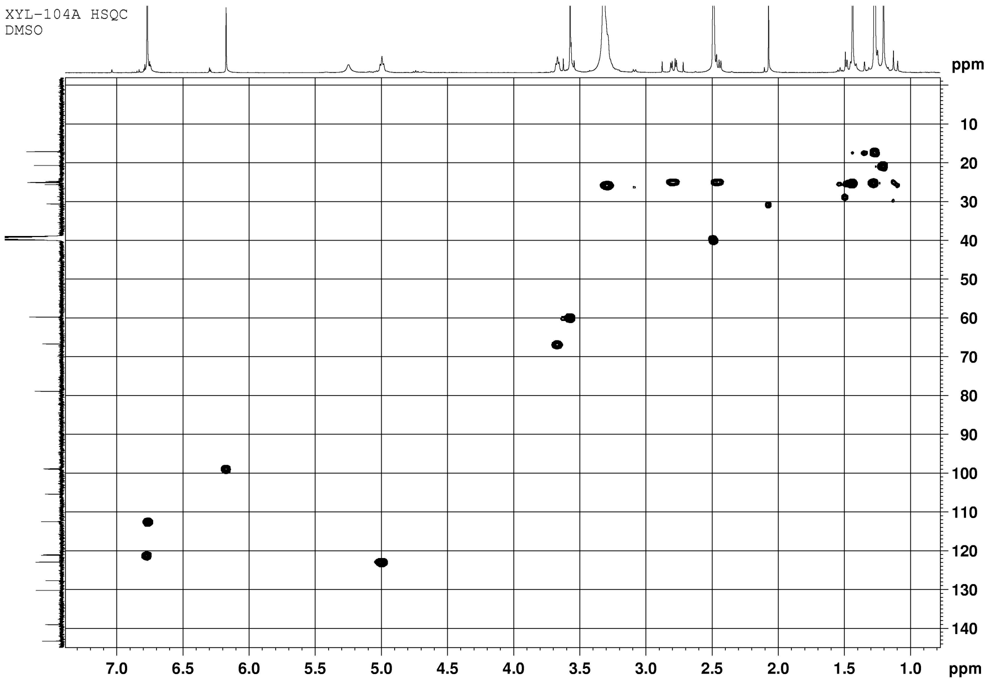

Figure S35. HSQC spectrum of compound **9**

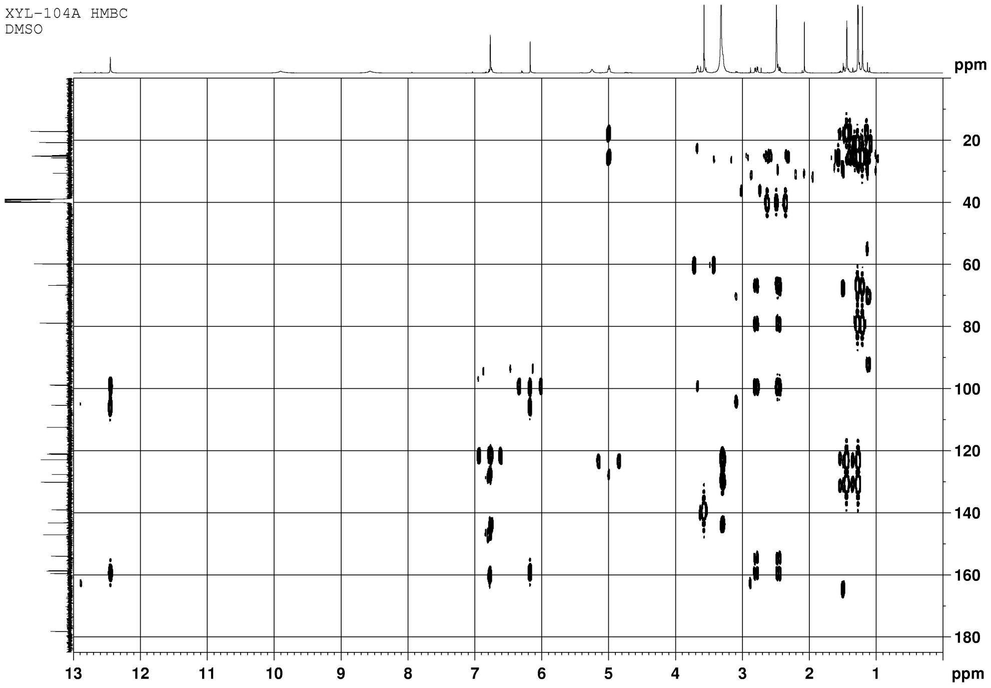

Figure S36. HMBC spectrum of compound **9**

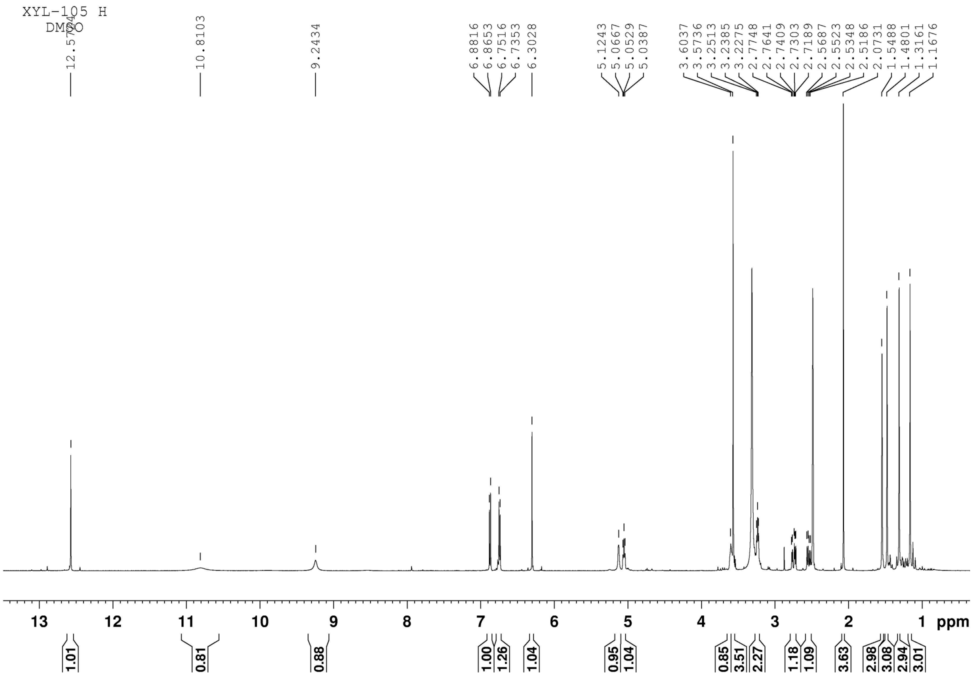

Figure S37. ^{1}H-NMR (500 MHz, DMSO-*d6*) spectrum of compound **10**

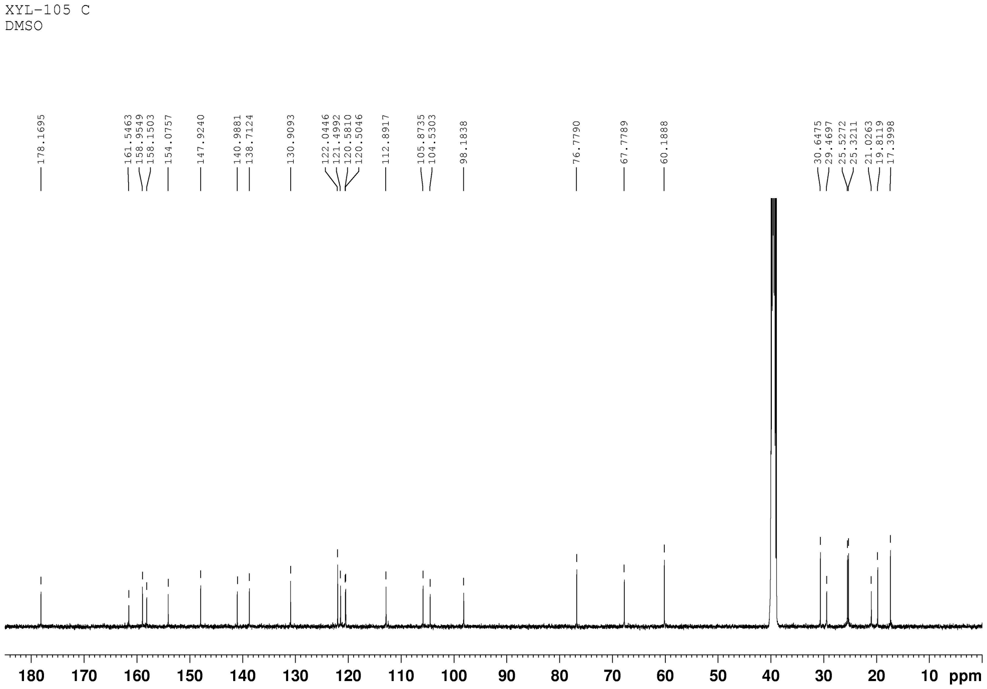

Figure S38. ^{13}C-NMR (125 MHz, DMSO-*d6*) spectrum of compound **10**

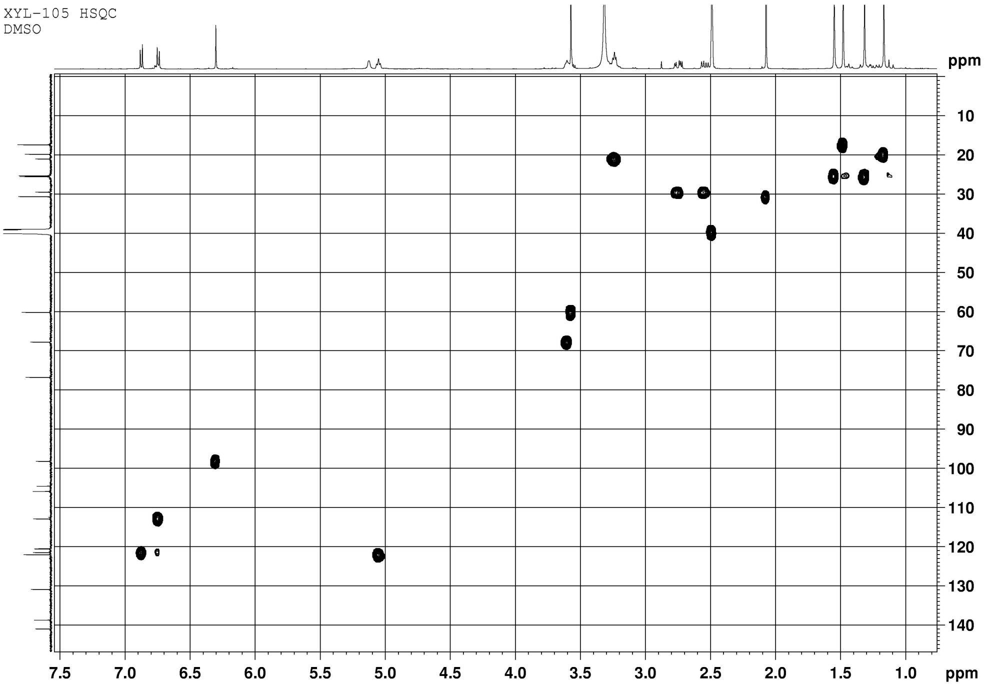

Figure S39. HSQC spectrum of compound **10**

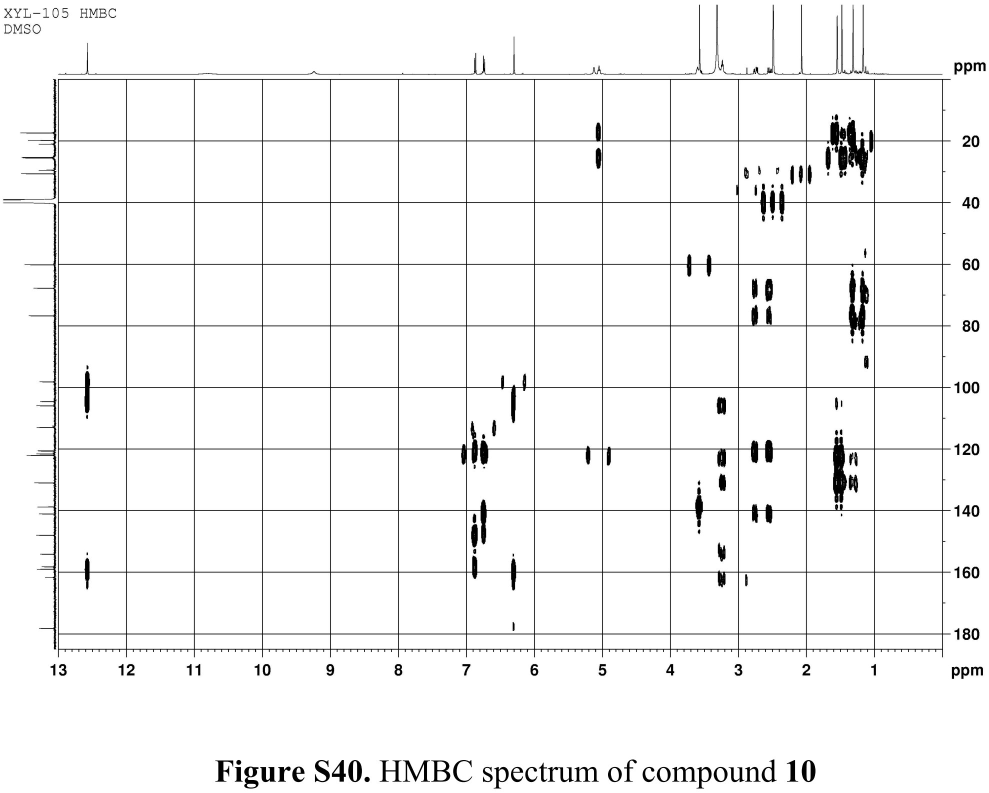

Figure S40. HMBC spectrum of compound **10**

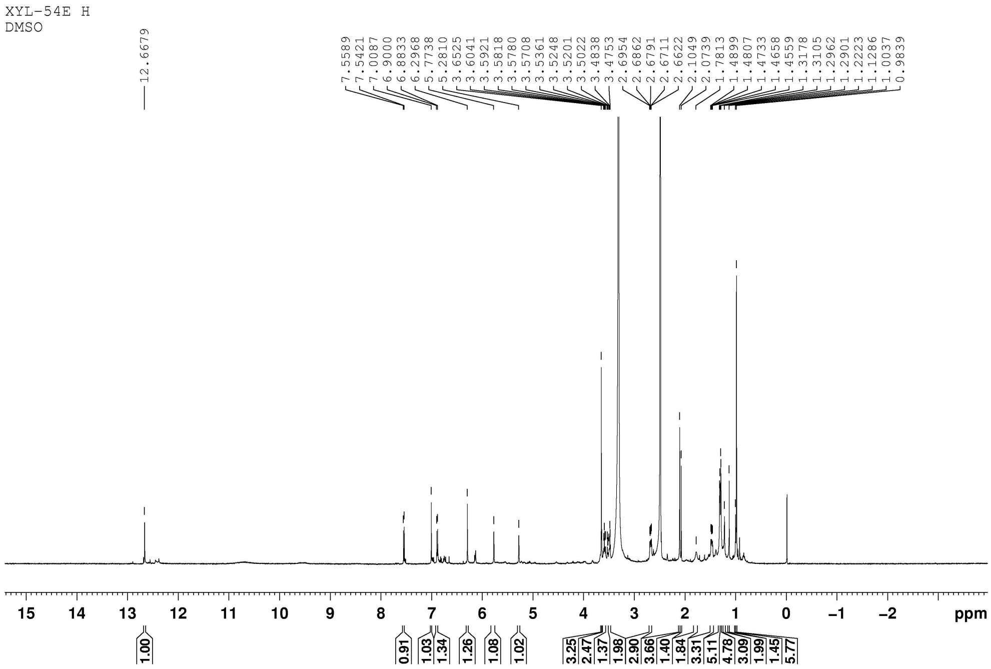

Figure S41. ^{1}H-NMR (500 MHz, DMSO-*d*6) spectrum of compound **11**

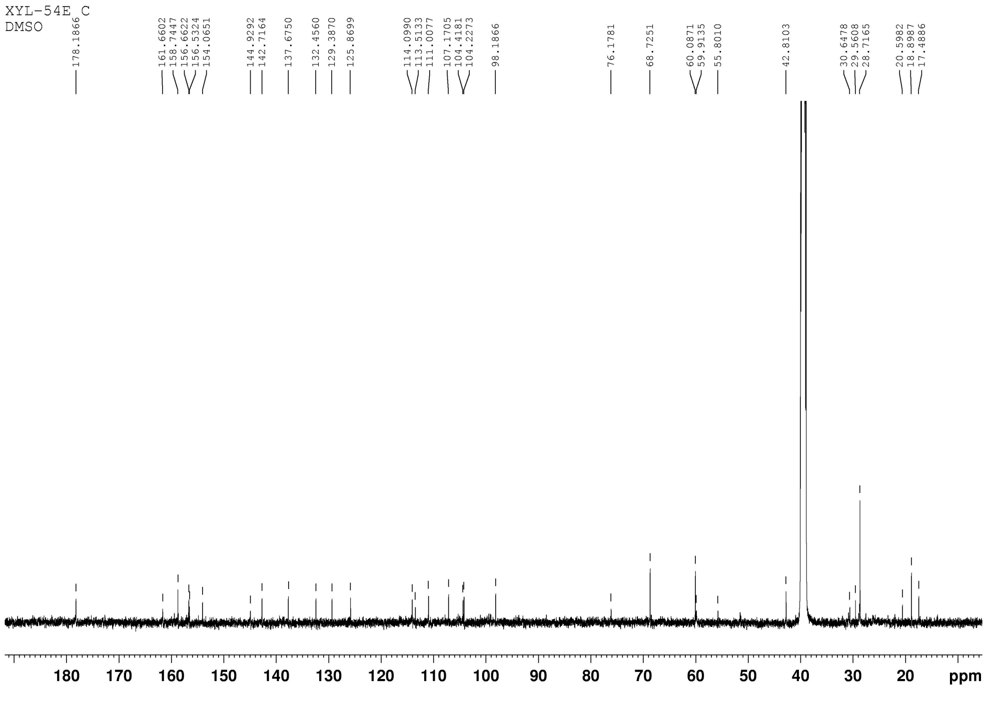

Figure S42. ^{13}C-NMR (125 MHz, DMSO-*d*6) spectrum of compound **11**

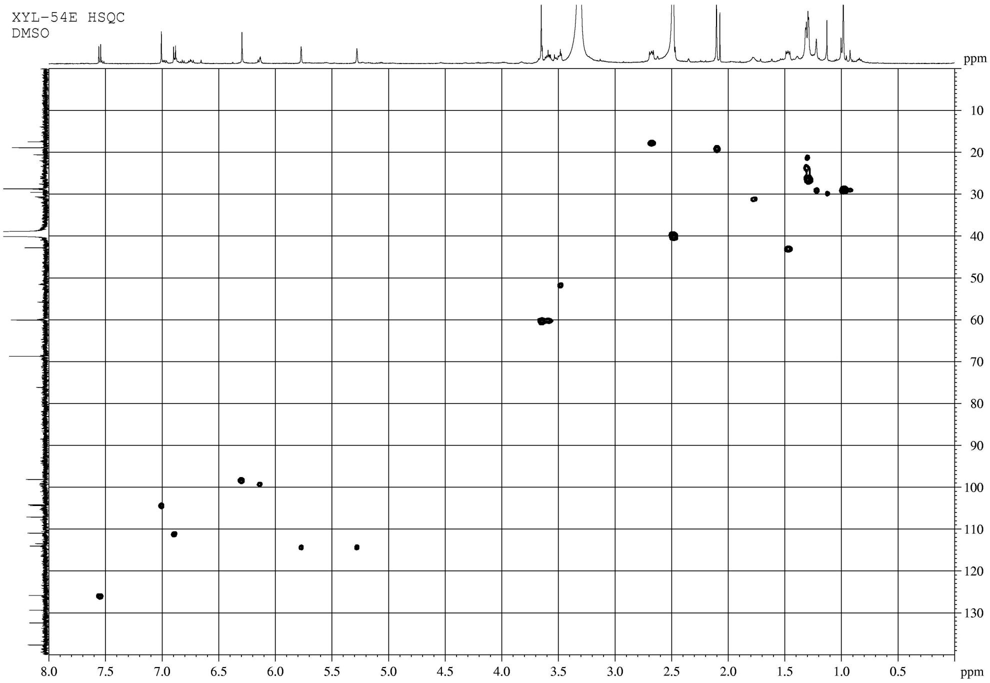

Figure S43. HSQC spectrum of compound **11**

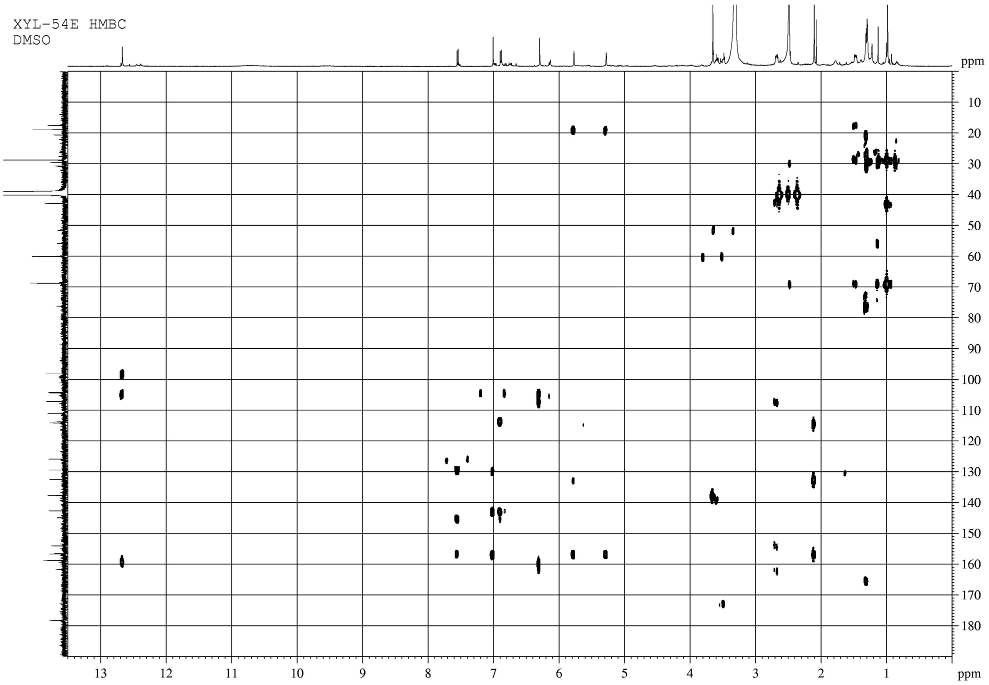

Figure S44. HMBC spectrum of compound **11**

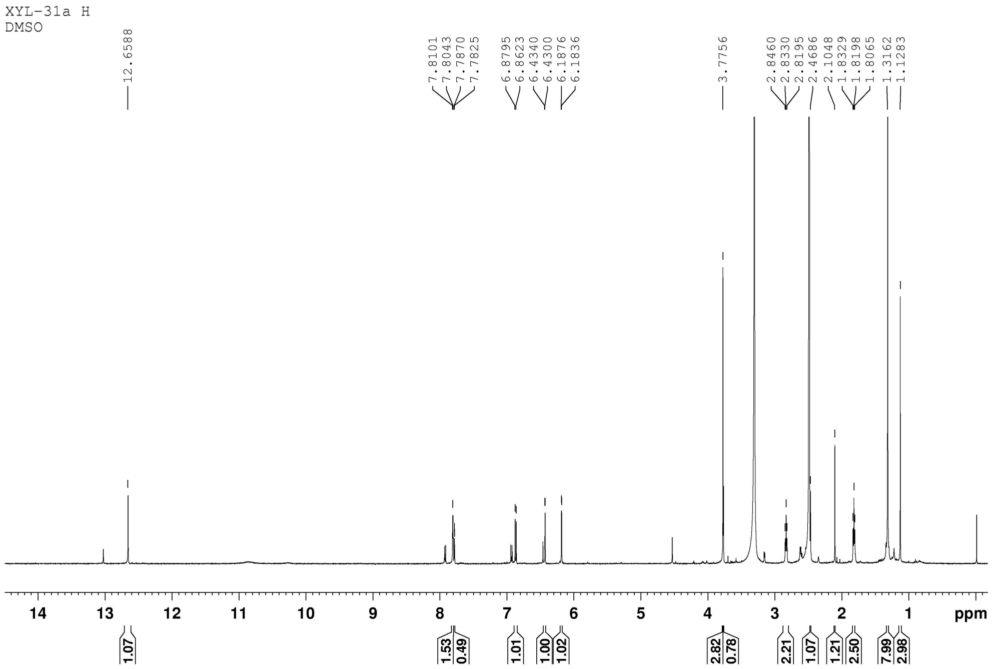

Figure S45. ^{1}H-NMR (500 MHz, DMSO-*d*6) spectrum of compound **12**

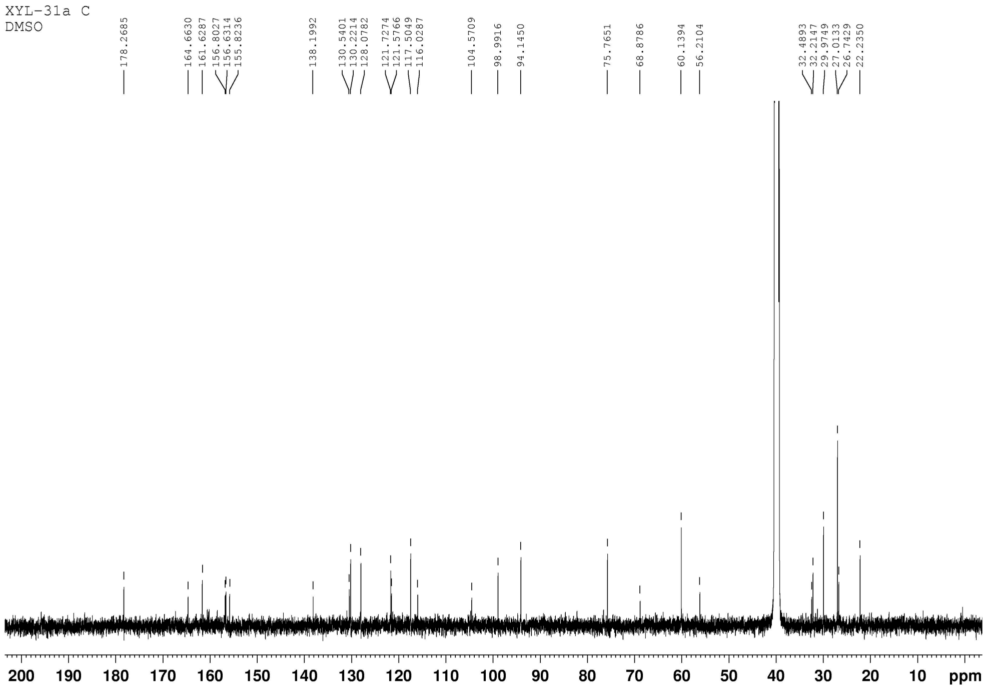

Figure S46. ^{13}C-NMR (125 MHz, DMSO-*d*6) spectrum of compound **12**

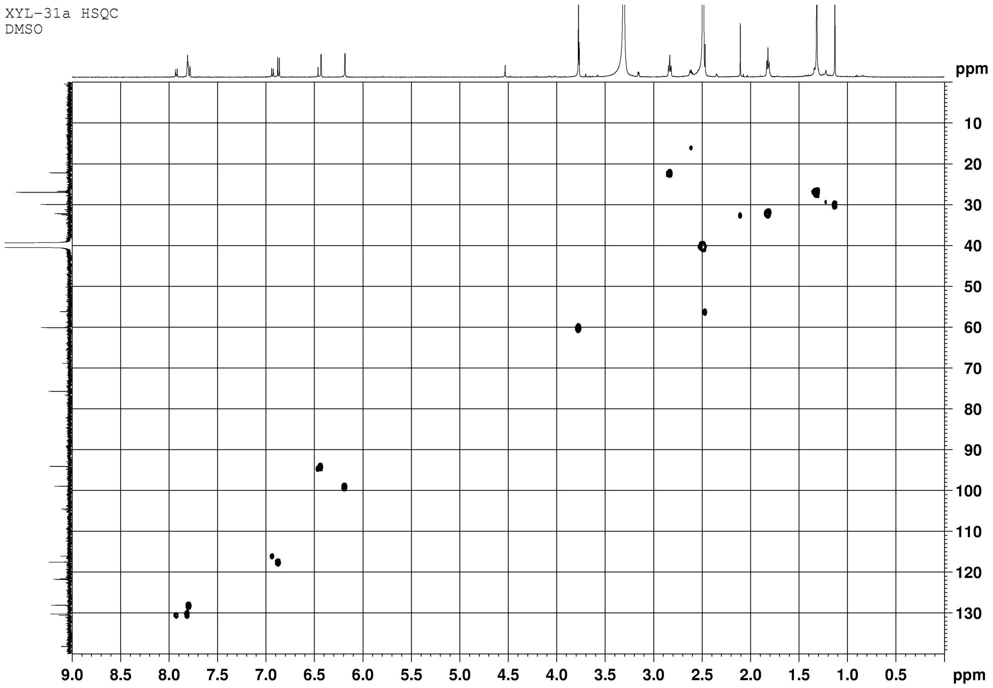

Figure S47. HSQC spectrum of compound **12**

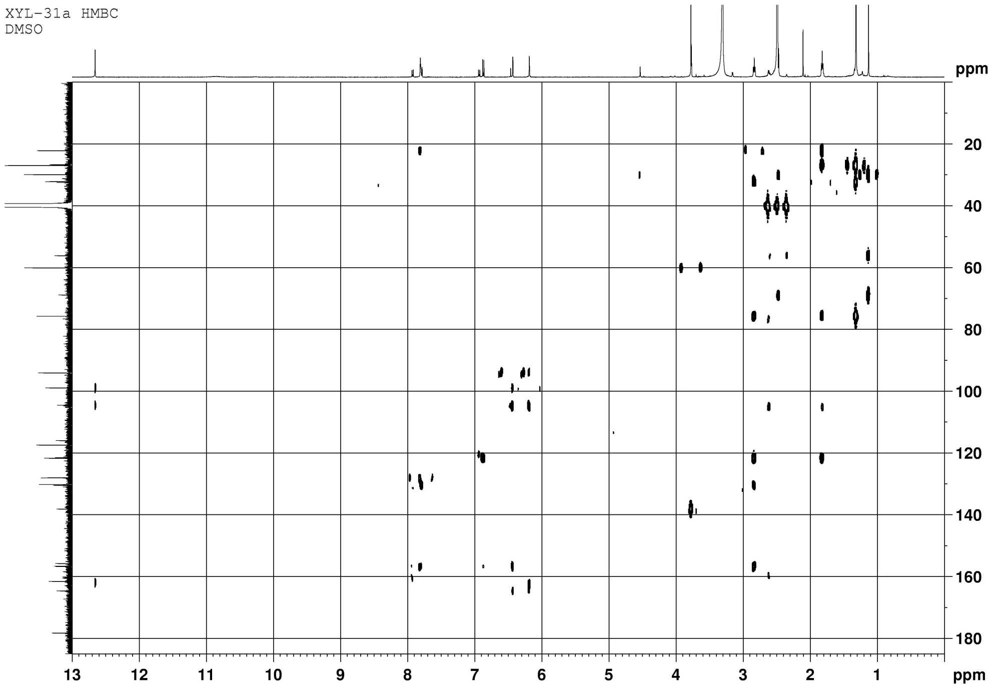

Figure S48. HMBC spectrum of compound **12**

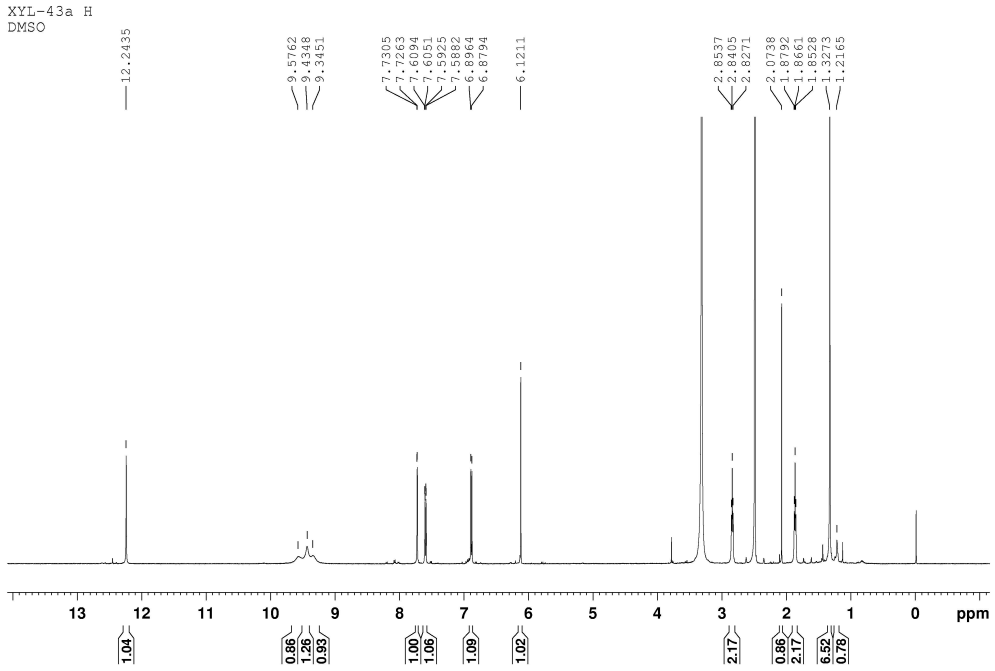

Figure S49. ^{1}H-NMR (500 MHz, DMSO-*d*6) spectrum of compound **13**

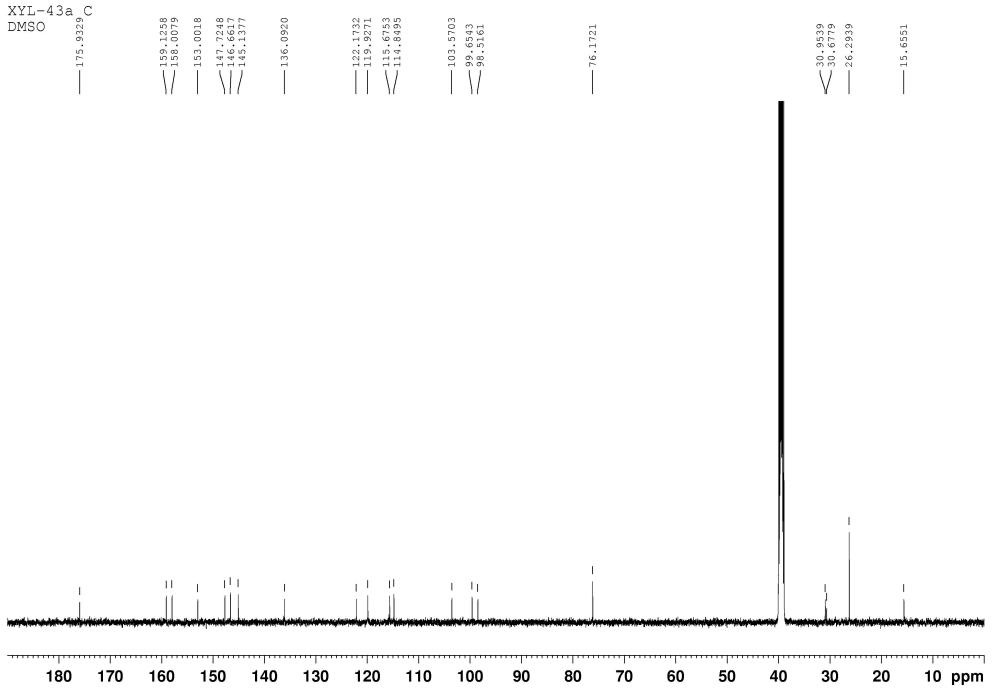

Figure S50. ^{13}C-NMR (125 MHz, DMSO-*d*6) spectrum of compound **13**

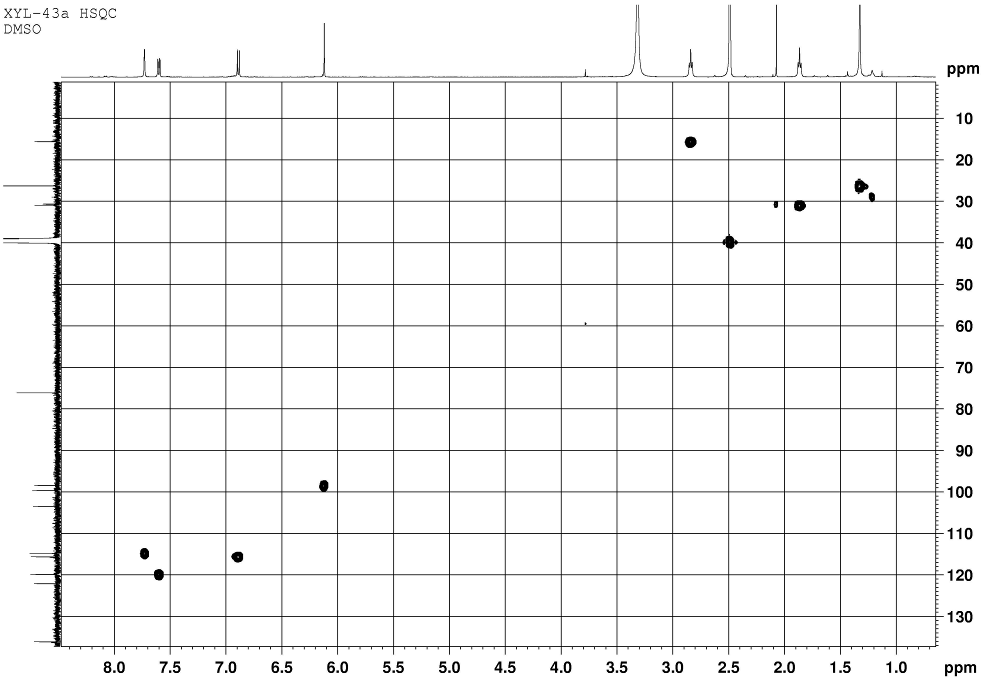

Figure S51. HSQC spectrum of compound **13**

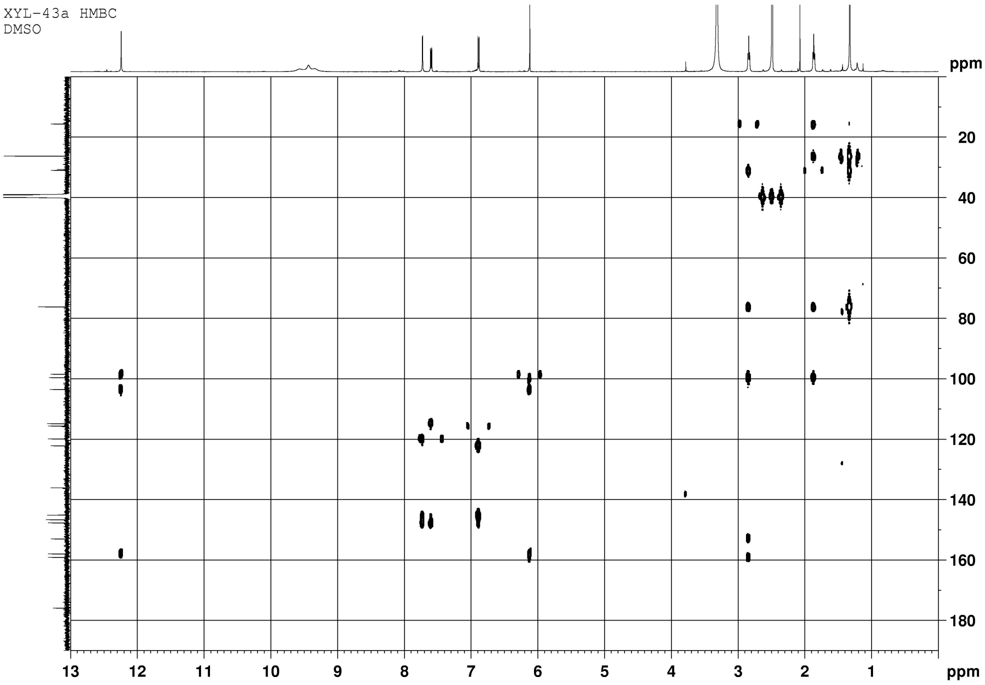

Figure S52. HMBC spectrum of compound **13**

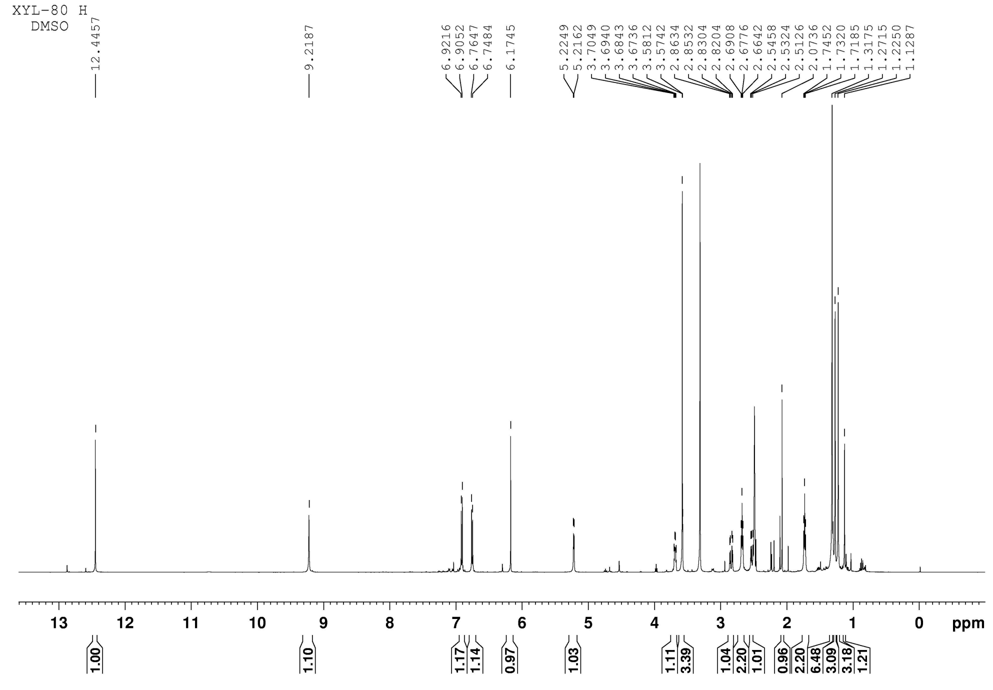

Figure S53. ^{1}H-NMR (500 MHz, DMSO-*d6*) spectrum of compound **14**

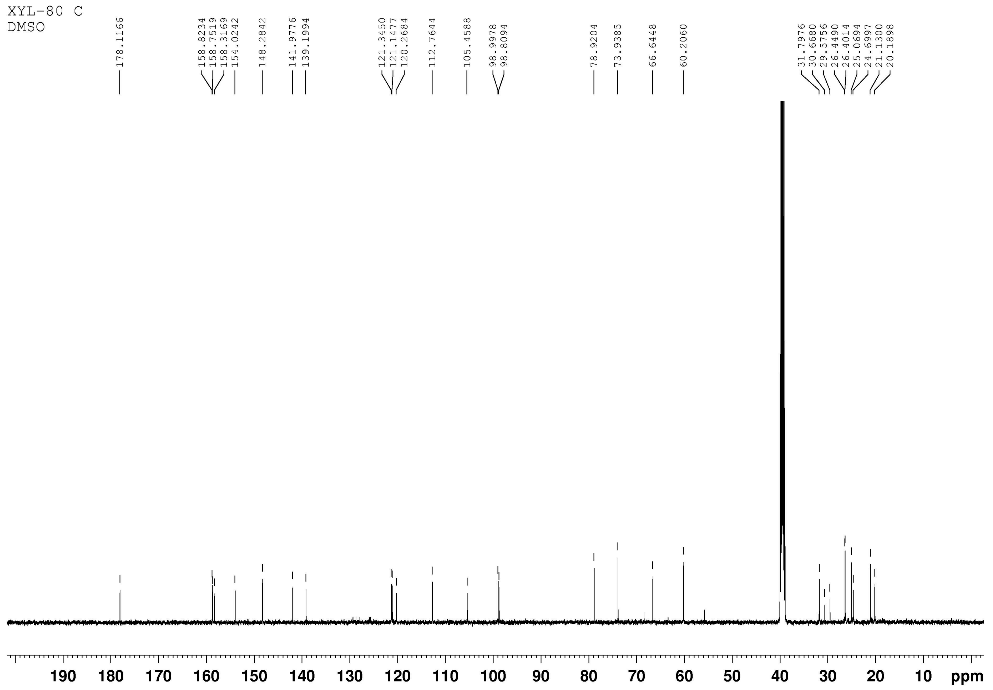

Figure S54. ^{13}C-NMR (125 MHz, DMSO-*d6*) spectrum of compound **14**

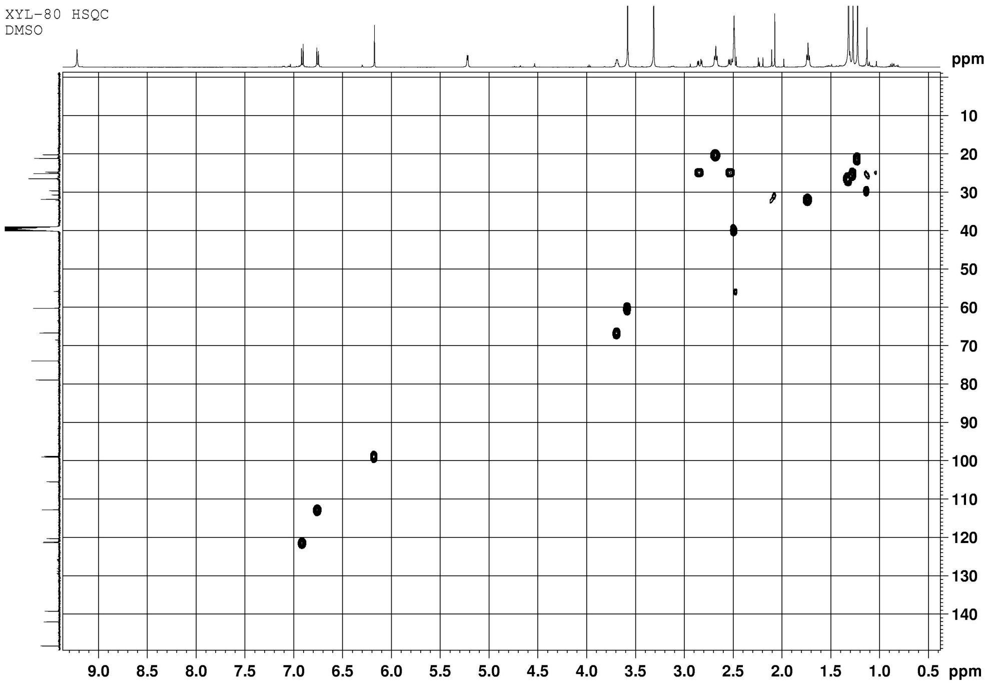

Figure S55. HSQC spectrum of compound **14**

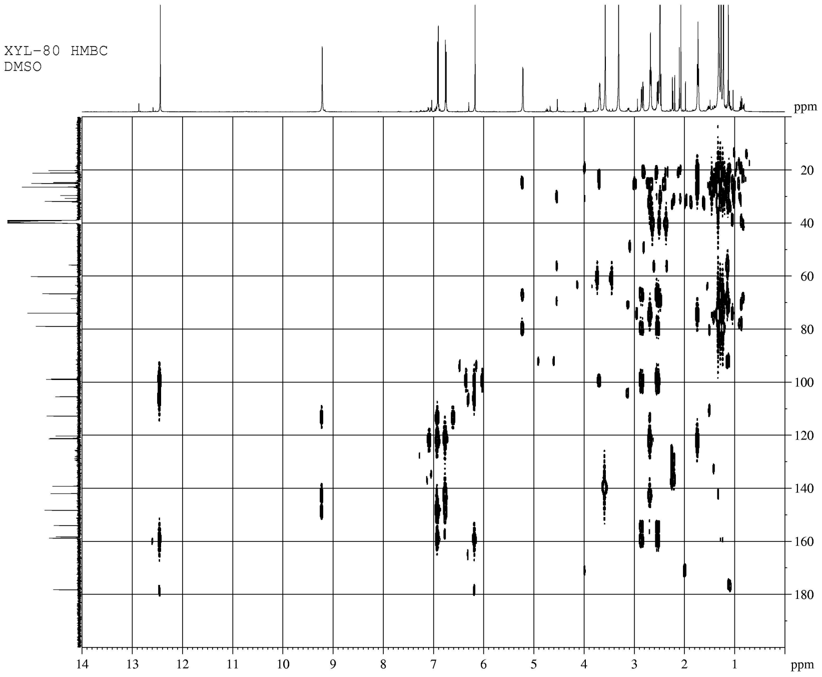

Figure S56. HMBC spectrum of compound **14**

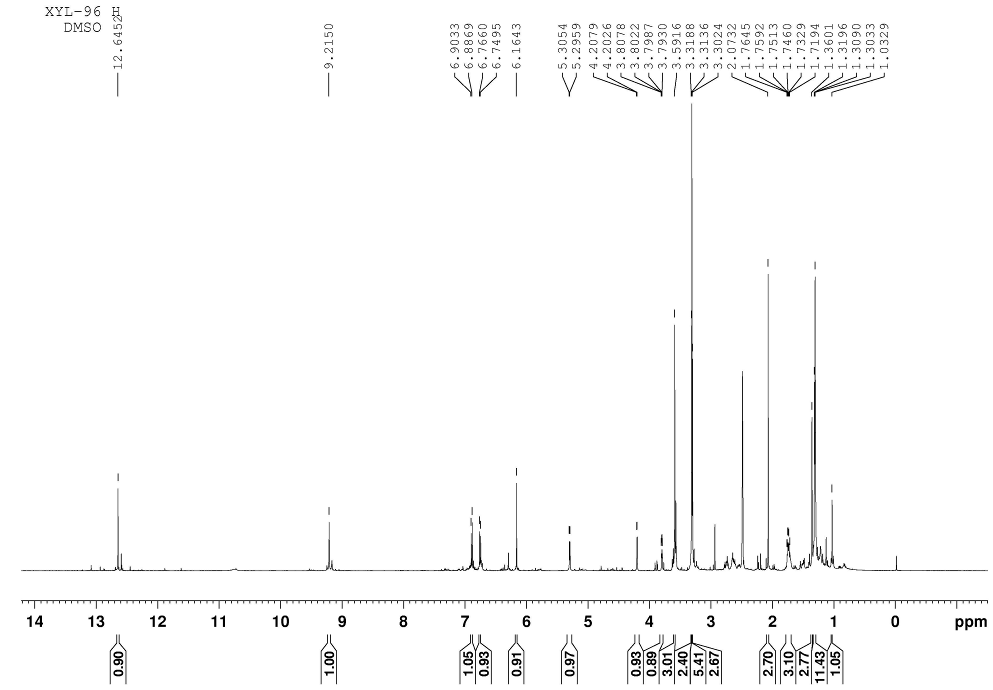

Figure S57. ^{1}H-NMR (500 MHz, DMSO-*d*6) spectrum of compound **15**

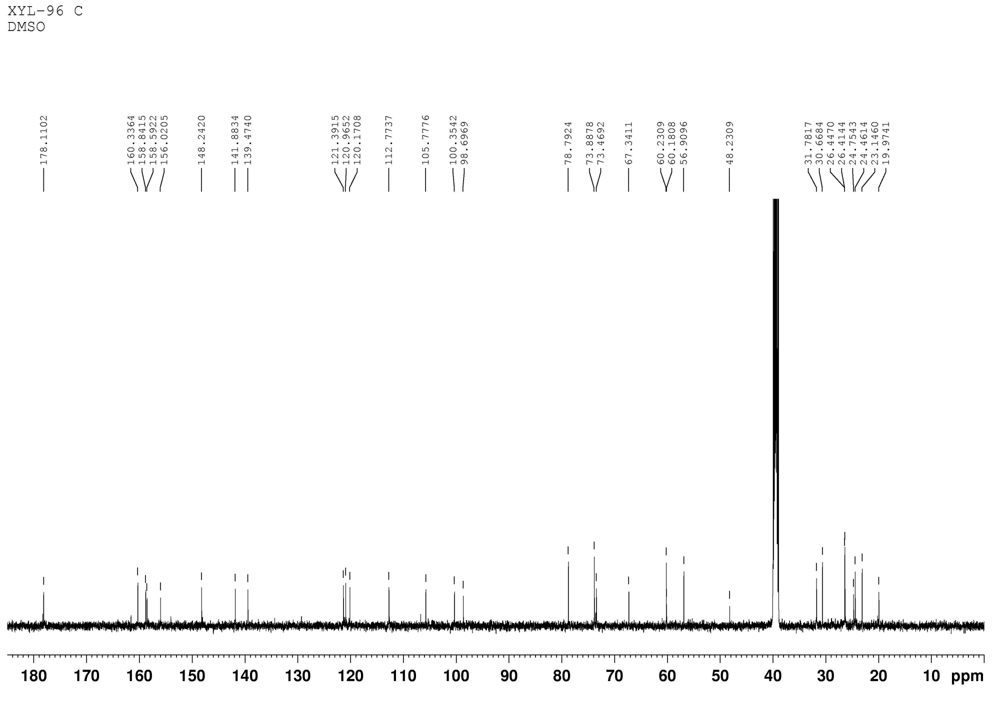

Figure S58. ^{13}C-NMR (125 MHz, DMSO-*d*6) spectrum of compound **15**

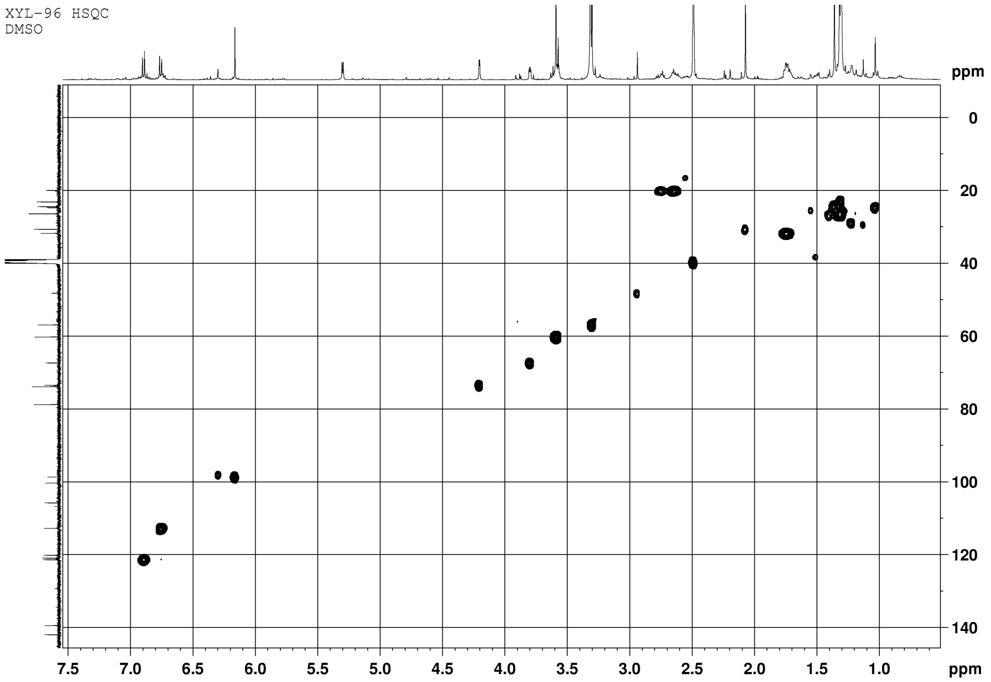

Figure S59. HSQC spectrum of compound **15**

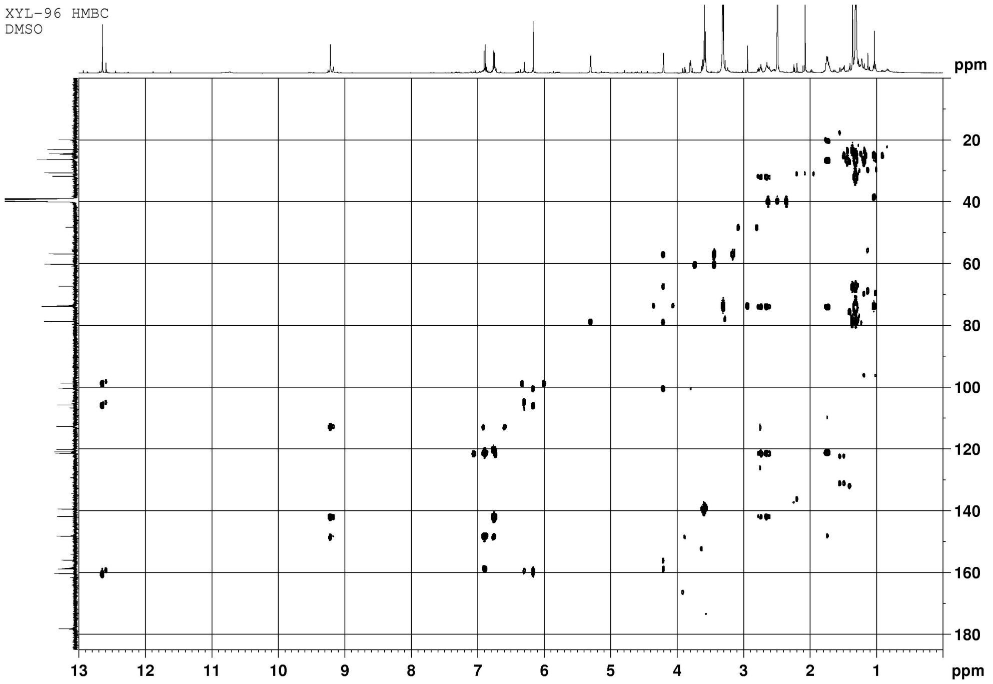

Figure S60. HMBC spectrum of compound **15**

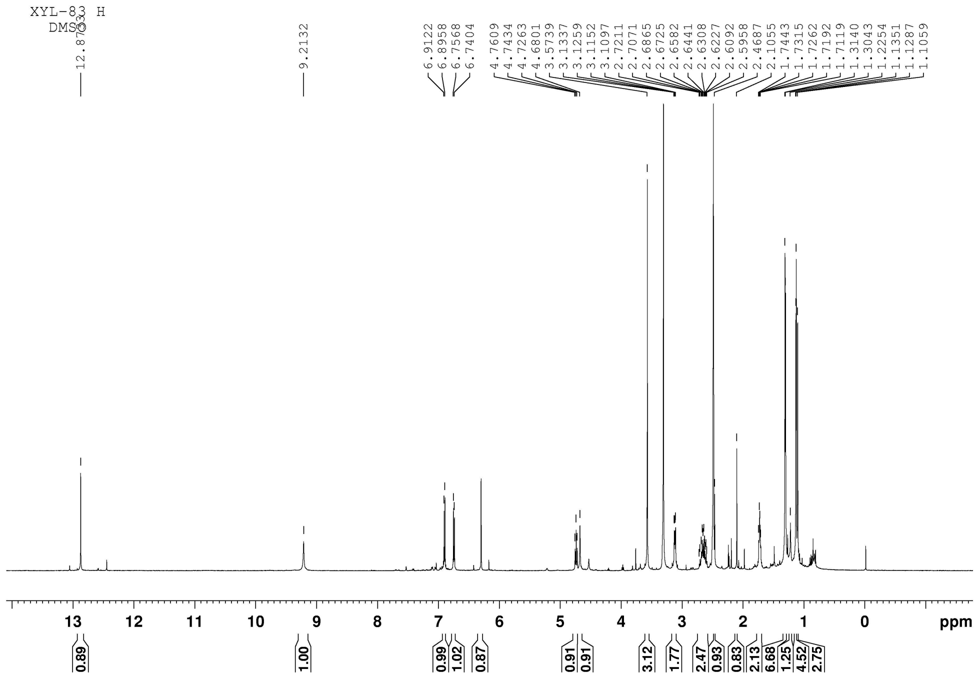

Figure S61. ^{1}H-NMR (500 MHz, DMSO-*d6*) spectrum of compound **16**

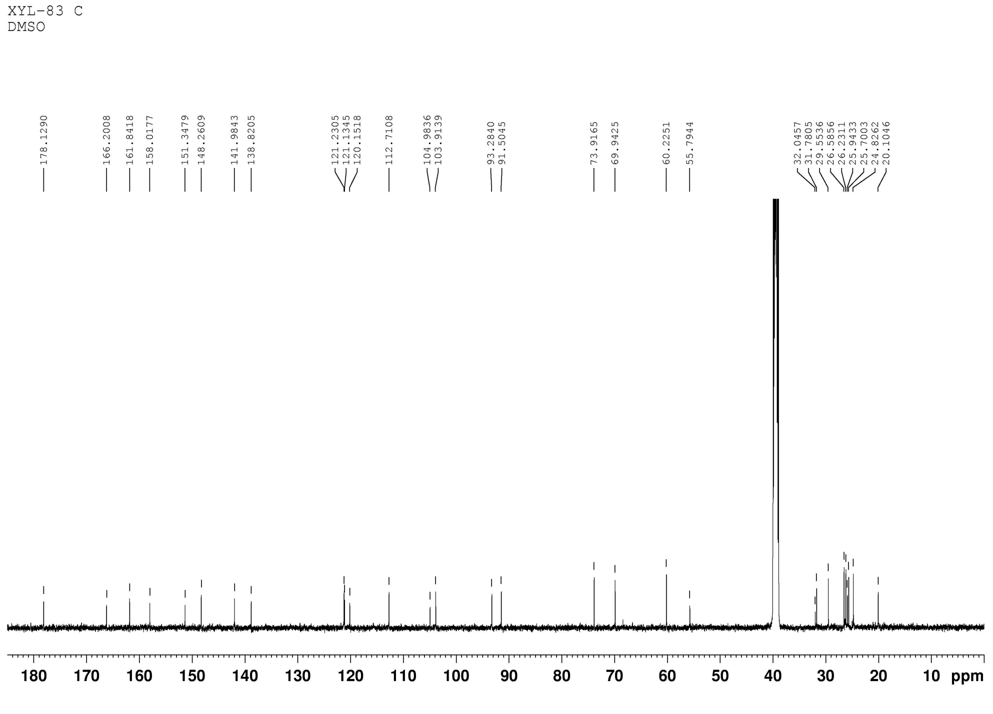

Figure S62. ^{13}C-NMR (125 MHz, DMSO-*d6*) spectrum of compound **16**

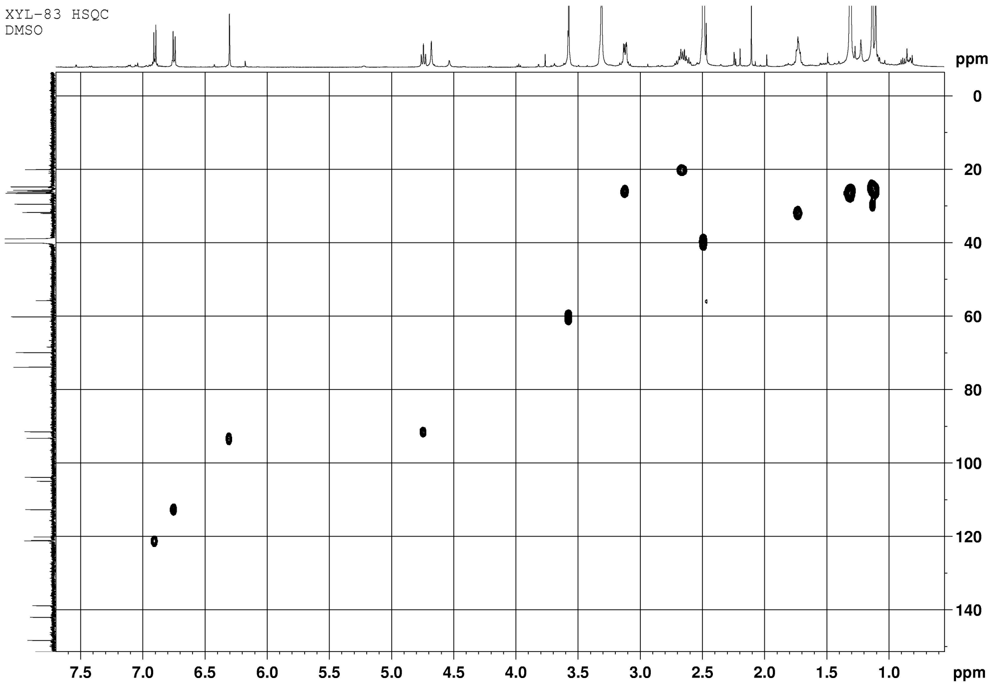

Figure S63. HSQC spectrum of compound **16**

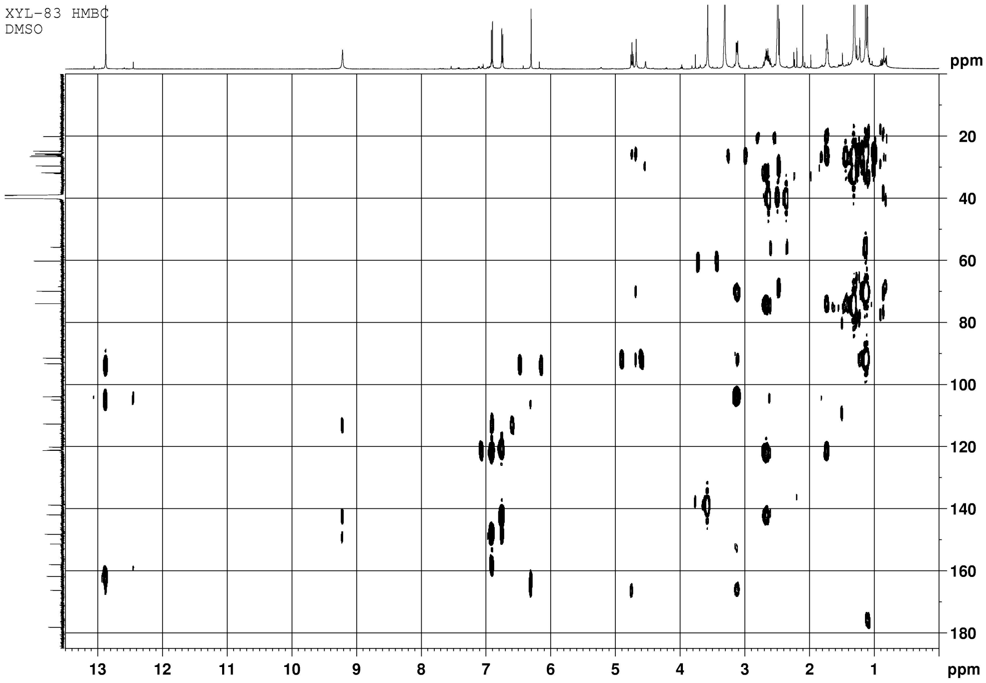

Figure S64. HMBC spectrum of compound **16**
